# Near-Absent Levels of Segregational Variation Suggest Limited Opportunities for the Introduction of Genetic Variation Via Homeologous Chromosome Pairing in Synthetic Neoallotetraploid *Mimulus*

**DOI:** 10.1534/g3.113.008441

**Published:** 2014-01-27

**Authors:** Jennifer L. Modliszewski, John H. Willis

**Affiliations:** Department of Biology, Duke University, Durham, North Carolina 27708

**Keywords:** *Mimulus*, neoallotetraploid, segregational variation, homeologous recombination, colchicine

## Abstract

Genetic variation is the fundamental medium of evolution. In allopolyploids, which are the product of hybridization and whole genome duplication, if homologous chromosomes always pair, then all descendants of a single diploid F_1_ hybrid lineage will be genetically identical. Contrarily, genetic variation among initially isogenic lineages is augmented when homeologous chromosomes pair; this added variation may contribute to phenotypic evolution. *Mimulus sookensis* is a naturally occurring, small-flowered allotetraploid derived from the large-flowered *Mimulus guttatus* and small-flowered *Mimulus nasutus*. Because diploid F_1_ hybrids between *M. guttatus* and *M. nasutus* have large flowers, phenotypic evolution post-polyploidization is implied in *M. sookensis*. Here, we present genetic and phenotypic analyses of synthetic neoallotetraploid *Mimulus* derived from a cross between *M. guttatus* and *M. nasutus*. Genetic marker data from S_2_ and BC_1N_ progeny suggest that chromosomes regularly pair with their homologous counterpart. By measuring the phenotype of synthetic neoallotetraploids, we demonstrate that polyploidization *per se* does not induce the small flowers of *M. sookensis*. Moreover, phenotypic measurements of synthetic allotetraploid F_2_s and S_4_ families suggest that rare homeologous recombination events have a negligible phenotypic effect in the first few generations. In total, the results are consistent with either exceedingly rare homeologous pairing and recombination or spontaneous fragment loss. The low levels of fragment loss and phenotypic variation in neoallotetraploids suggest that homeologous recombination after polyploidization is not a major mechanism of phenotypic evolution in *M. sookensis*. Rather, it may be that spontaneous mutations or epigenetic changes after allopolyploidization have driven phenotypic evolution in *M. sookensis.*

Genetic variation within a species is essential for evolution by natural selection and genetic drift. In plants, polyploidization, or whole genome duplication, is a common and instantaneous mode of speciation (Dobzhansky 1937; Masterson 1994; Otto and Whitton 2000; Blanc and Wolfe 2004; Coyne and Orr 2004; Cui *et al.* 2006). A unique aspect of polyploid speciation is that species formation can involve only a single individual, creating an extreme genetic bottleneck. As a consequence of this, when allopolyploids form from diploid F_1_ hybrids, it is possible that all descendants of a single individual will be genetically identical, with the exception of spontaneous mutations and ploidy-mediated genetic changes (Comai 2005; Soltis *et al.* 2003; Chen 2007; Doyle *et al.* 2008). Spontaneous mutations and method of polyploid formation aside, the extent to which descendent lineages are genetically identical depends on the pattern of chromosome pairing at meiosis (Stebbins 1971; Comai 2005; Ramsey and Schemske 2002). Allopolyploids will lack genetic variation if the homologous chromosomes from within each diploid progenitor species pair faithfully during meiosis (Figure 1, A and C and Table 1). However, if any of the homeologous chromosomes from different diploid progenitors pair with one another, then multiple gamete types will be produced, resulting in genetically variable offspring (Figure 1, B and D and Table 1).

**Figure 1:**
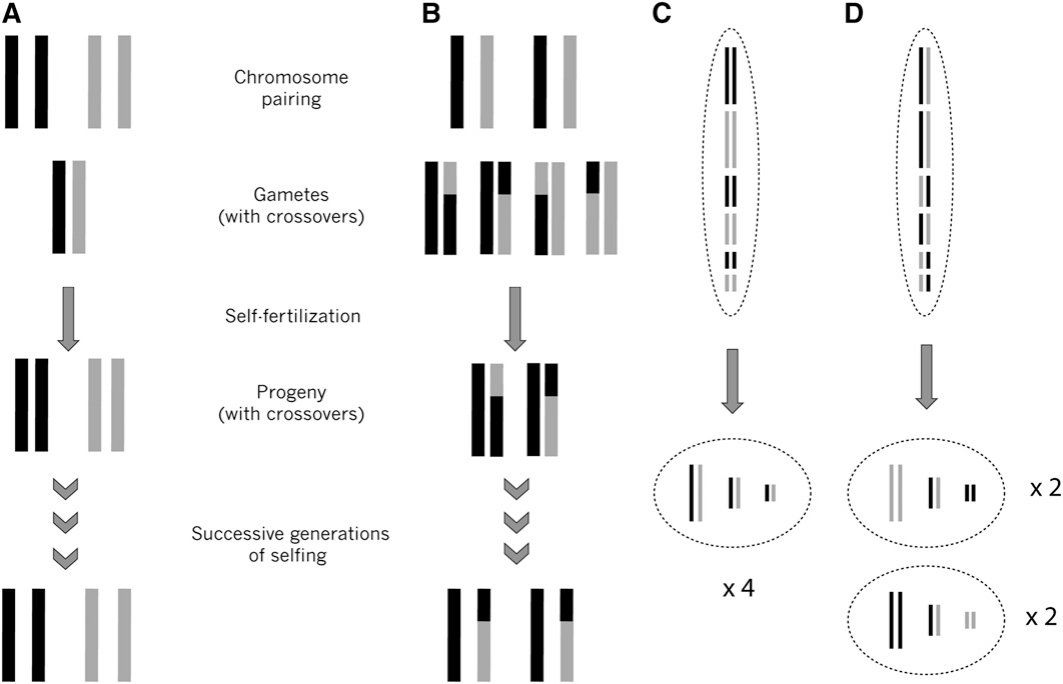
Illustration of expectations under homologous or homeologous chromosome pairing. Chromosome pairings of the same color (black–black or gray–gray) are homologous and chromosomes of different colors (gray–black) are homeologous. (A) Homologous pairing and subsequent fixed heterozygosity. (B) Homeologous pairing and novel genetic variation created by segregation and recombination. (C) Homologous pairing and independent assortment do not further contribute to genetic variation. (D) Homeologous pairing and independent assortment further contribute to genetic variation among offspring.

**Table 1:** Expected gametic ratios, genotypic frequencies, and observed apparent heterozygosity in F_2_ and BC_1_ progeny in five models of chromosome pairing

**Chromosome Pairing Model**	**Inheritance**	**Gametic Ratios in F_1_ Hybrid**	**Genotype Frequencies**		**Expected Apparent Heterozygosity**	
			**F_2_ Progeny**	**BC_1_ Progeny**	**F_2_ Progeny**	**BC_1_ Progeny**
Homolog–homolog (autosyndesis)	Disomic	0 AA:1 Aa:0 aa	All AAaa	All Aaaa	1.00	1.00
Homolog–homeolog (allosyndesis)	Disomic	1AA:2Aa:1aa	1 AAAA:4 AAAa:6 AAaa:4 Aaaa:1 aaaa	1 AAaa:2 Aaaa:1 aaaa	0.88	0.75
Random chromosome assortment/random bivalent	Disomic or tetrasomic	1AA:4Aa:1aa	1 AAAA:8 AAAa:18 AAaa:8 Aaaa:1 aaaa	1 AAaa:4 Aaaa:1 aaaa	0.94	0.83
Random chromatid assortment	Tetrasomic	3AA:8Aa:3aa	9 AAAA:48 AAAa:82 AAaa:48 Aaaa:9 aaaa	3 AAaa:8 Aaaa:3 aaaa	0.91	0.79
Maximal equational segregation	Tetrasomic	2AA:5Aa:2aa	4 AAAA:20 AAAa:33 AAaa:20 Aaaa:4 aaaa	2 AAaa:5 Aaaa:2 aaaa	0.90	0.78

Expected apparent heterozygosity refers to the proportion of individuals expected to have a microsatellite band from each parent, even if they are not balanced heterozygotes (*i.e.*, Aaaa individuals). For F_2_ progeny, both homozygous classes are combined together to calculate the expected apparent heterozygosity.

Homeologous chromosome pairing at meiosis affects genetic variation in gametes and subsequent progeny by segregation, which results in genetic variation among gametes at a single locus, and by independent assortment, which results in genetic variation within a gamete by random alignment of all chromosome pairs during metaphase I of meiosis (Figure 1D). More specifically, if one considers a single locus, an allotetraploid individual with the genotype AAaa will only produce Aa gametes across all loci if homologous chromosomes pair, a phenomenon known as fixed heterozygosity (Ramsey and Schemske 2002). If homeologous chromosomes pair, then AA, Aa, and aa gametes will be produced, resulting in five possible zygotic genotypes (AAAA, AAAa, AAaa, Aaaa, and aaaa) and genetically variable offspring. The ratio of gamete types (*i.e.*, AA, Aa, aa) for a single locus produced by homeologous chromosome pairing depends on whether recombination occurs between the locus and the centromere (Table 1) (Haldane 1930; Mather 1935). If recombination does not occur, then random chromosome assortment, as modeled by Gregory 1914 and Muller 1914, and segregation of chromosomes will produce an expected gametic ratio of 1AA:4Aa and 1aa (Table 1) (Muller 1914). If recombination occurs between the locus and the centromere, as first proposed by Haldane (1930), then the expected gametic ratio becomes 2AA:5Aa:2aa (random chromatid assortment) (File S1, Figure S1 and Table 1) (Mather 1935, 1936; Burnham 1962).

Although chromosome pairing models are useful for predicting expected gametic ratios and genotype frequencies, adherence to expectations will vary among loci according to three conditions: the proximity of the locus to the centromere; the particular chromosome pair being considered; and how strictly a particular chromosome associates with its homolog. The last condition, the degree of preferential pairing (Sybenga 1994), refers to the fact that chromosomes may form any combination of homologous bivalents, homeologous bivalents, or quadrivalents; this has been observed in autotetraploid *Rorippa* species and *Crassostrea gigas* (Curole and Hedgecock 2005; Stift *et al.* 2008). When only homologous bivalents form, chromosomes are said to preferentially pair.

By measuring the phenotype of polyploid F_2_ populations at single-locus traits, early plant breeders, focusing mainly on autotetraploids, were able to reveal that gametic ratios vary by locus in accordance with random chromosome and random chromatid pairing models (Blakeslee *et al.* 1923; Lindstrom and Humphrey 1933; Fisher 1941; Doyle 1973; Little 1945, 1958). Surprisingly, in allopolyploids homeologous chromosome pairing and recombination have also been inferred (Crane and Darlington 1932; Sansome 1933; Gerstel and Phillips 1957, 1958; Gerstel 1960). More recently, it has been shown in *Brassica napus* that allopolyploid lines that began as a genetically homogeneous population can accumulate genetic variation among lineages by the first generation after allopolyploidization as a result of homeologous recombination; most importantly, this genetic variation is associated with variation in flowering time (Schranz and Osborn 2000, 2004; Gaeta *et al.* 2007; Szadkowski *et al.* 2010).

Homeologous recombination in *Gossypium* (Salmon *et al.* 2010) and translocations and reciprocal aneuploidy in *Tragopogon* (Lim *et al.* 2008; Chester *et al.* 2012) may create genetic variation among initially isogenic lines, but these changes have not been linked to phenotypic variation. Gene expression in allopolyploids has also been reported to vary from expectations based on an additive model, suggesting that epigenetic changes may also play a role in phenotypic divergence between polyploid and diploid progenitor (Liu *et al.* 2001; Wang *et al.* 2006; Flagel *et al.* 2008; Chaudhary *et al.* 2009; Rapp *et al.* 2009). Of the studies that have examined genetic and epigenetic changes after polyploidization, surprisingly few have made a concerted effort to record phenotypic variation of synthetic and natural allopolyploids while also examining genomic changes (Comai *et al.* 2000; Anssour *et al.* 2009; Matsushita *et al.* 2012). In synthetic autotetraploid *Heuchera grossulariifolia*, polyploidization appears to cause an immediate phenotypic shift toward trait values that may have been beneficial during establishment, although flower size in synthetic *Heuchera grossulariifolia* is large, unlike in natural tetraploid *Heuchera grossulariifolia* (Oswald and Nuismer 2011). Thus, aside from the results in *Brassica napus*, little is known about how the multitude of genetic and epigenetic changes observed in synthetic polyploids may lead to the phenotype observed in a naturally occurring or crop allopolyploid.

*Mimulus sookensis* (Benedict *et al.* 2012) is an allotetraploid derivative of the large-flowered, predominately out-crossing *Mimulus guttatus* and the small-flowered, highly selfing *Mimulus nasutus* (Sweigart *et al.* 2008; Modliszewski and Willis 2012). *M. sookensis* is small-flowered, similar to *M. nasutus* (Benedict *et al.* 2012), but diploid F_1_ hybrids are somewhat intermediate and phenotypically similar to *M. guttatus* because of partial dominance of *M. guttatus* floral characters (Fishman *et al.* 2002); this suggests that phenotypic evolution occurred following polyploidization. A genetic linkage map constructed from a *M. guttatus* × *M. nasutus* F_2_ population establishes that chromosome pairing and recombination occur in diploid F_1_ hybrids between *M. guttatus* and *M. nasutus* (Fishman and Willis 2001). Consequentially, the homeologous chromosomes of *M. guttatus* and *M. nasutus* are predicted to be able to readily pair when placed together in an allopolyploid genome, possibly leading to homeologous recombination and rapid recapitulation of the phenotype of natural *M. sookensis* in synthetic allopolyploids derived from *M. guttatus* and *M. nasutus*. Although the chromosomes of *M. guttatus* and *M. nasutus* are capable of pairing in diploid F_1_s, in an allotetraploid the two chromosomes of *M. guttatus* may be more likely to pair with one another, and likewise for the two homologous *M. nasutus* chromosomes. For this reason, it is unknown how the chromosomes of allotetraploid *Mimulus* will pair.

Here, our broad objective was to determine how chromosome pairing following polyploidization may affect phenotypic evolution in *M. sookensis*. We first asked if there was evidence for homeologous chromosome pairing and recombination, using genetic marker data from artificially synthesized neoallotetraploid *Mimulus* derived from diploid *M. guttatus* and *M. nasutus*. We then assessed the immediate phenotypic effects of polyploidization by measuring the floral phenotype of neoallotetraploid *Mimulus* and comparing it to diploid *M. guttatus* and *M. nasutus*, diploid hybrids, and *M. sookensis*. Finally, to shed light on the evolutionary processes that have shaped flower size in naturally occurring *M. sookensis*, we asked if homeologous recombination may facilitate an enhanced rate of phenotypic evolution by contributing to phenotypic variation. We address this question by comparing the variance of quantitative traits in F_1_ and F_2_ hybrids and S_4_ families. Both the genetic marker data and phenotypic measurements will reveal any heritable genetic variation in newly formed synthetic allotetraploid *Mimulus*, which natural selection may act on.

## Materials and Methods

### Generation of synthetic tetraploid lines

To generate synthetic tetraploid lines, diploid seed from *M. guttatus* IM62, *M. nasutus* SF, and diploid F_1_ hybrid seed generated by reciprocally crossing *M. guttatus* IM62 and *M. nasutus* SF was soaked in a 0.0001%, 0.001%, and 0.01% colchicine solution for 6 hr. Both *M. guttatus* IM62 and *M. nasutus* SF are highly inbred lines (>12 generations), greatly reducing the likelihood of residual heterozygosity in diploid and autotetraploid lines. Because only F_1_ hybrid seeds were transformed into polyploids at the aforementioned concentrations, subsequent treatments of *M. nasutus* SF and *M. guttatus* IM62 were performed with concentrations of 0.05% and 0.1% colchicine. After the soak, seeds were rinsed ∼10 times using distilled water and pipetted onto premoistened Fafard 4p potting medium. To identify tetraploid lines, plants were screened using flow cytometry once sufficient leaf and bud material was present, as described previously (Modliszewski and Willis 2012), using NIB chopping buffer (Bino *et al.* 1992). To increase the efficiency of the screening process, four individuals and an internal control (*Petunia* × *hybrida* “Wave”) were processed simultaneously. If tetraploid peaks were evident, then all four individuals were run separately to identify the tetraploid individual(s).

**Figure 2:**
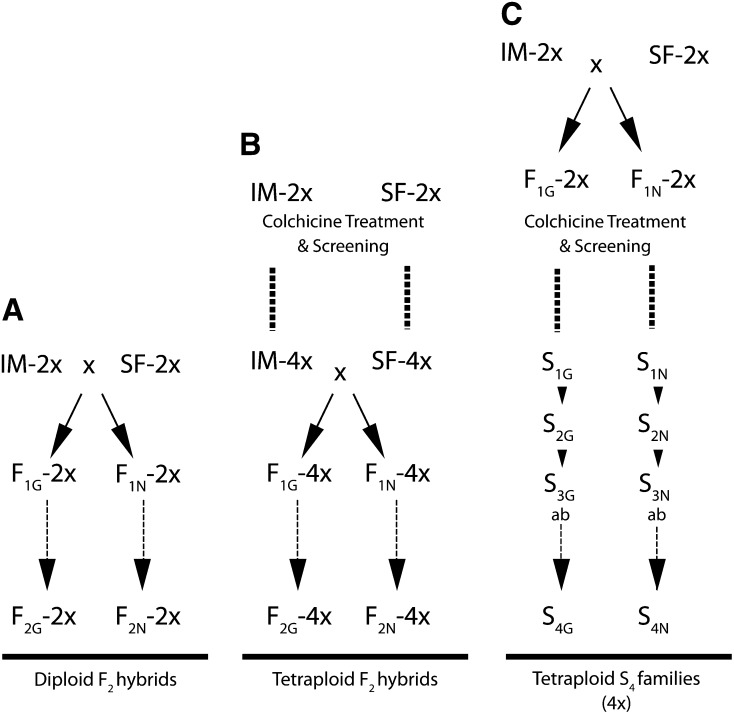
Crossing design for phenotypic analyses. (A) Diploids. (B) Synthetic F_2_ tetraploids. (C) Synthetic tetraploid S_4_ families. 2x, diploid; 4x, tetraploid; IM, *M. guttatus* IM62; SF, *M. nasutus* SF; subscripted “G” and “N” are used to indicate that *M. guttatus* (G) or *M. nasutus* (N) was the maternal parent. All S-lines (S_1_–S_4_) are tetraploid. S_2_ lines were used in genetic marker analysis; S_3_ lines were split into “a” and “b” subfamilies to test for maternal effects in the S_4_ families. BC_1N_ lines were generated by backcrossing F_1N_-4x to SF-4x.

Two forms of synthetic neoallotetraploid lines and one diploid F_2_ population were generated (Figure 2). For the purpose of clarity, we hereafter refer to the combination of genotype and ploidy as a class (for example, IM-2x indicates diploid *M. guttatus* IM62), with reciprocal hybrid classes combined, unless indicated with G or N, which are used to designate the maternal parent (for example, F_1G_-4x is used to indicate tetraploid F_1_ hybrids with *M. guttatus* IM62 as the maternal parent, whereas F_1_-4x plants are tetraploid F_1_ hybrids with either *M. nasutus* SF or *M. guttatus* IM62 as the maternal parent). Synthetic neotetraploid *Mimulus* lines are referred to as “neoallotetraploids” (*e.g.*, F_1_-4x, F_2_-4x, S_4_ families) or “neoautotetraploids,” as appropriate. Neoallotetraploid lines were first created by synthesizing neoautotetraploid parents [*M. guttatus* IM62 (IM-4x) and *M. nasutus* SF (SF-4x)] and reciprocally crossing them to create F_1_ neoallotetraploids (F_1_-4x). First-generation backcross progeny (BC_1N_) were generated for genetic marker analysis by crossing the resulting F_1N_-4x neoallotetraploid to neoautotetraploid *M. nasutus* SF; the F_1N_ plant was used as the maternal parent in this cross. Reciprocal F_1_ neoallotetraploids were also selfed to create two F_2_ populations for use in phenotypic analyses; reciprocally crossed populations were treated as a single population for most analyses (Figure 2B). Neoallotetraploids were also generated by treating diploid F_1_ seed from reciprocal crosses between *M. guttatus* IM62 and *M. nasutus* SF with colchicine. The progeny from two reciprocally crossed neoallotetraploid individuals were made into 48 family lines for each cross direction (S-lines), which were then selfed for three generations to create two groups of S_4_ families, S_4G_ and S_4N_ (Figure 2C), which were used in the phenotypic analysis. Individuals from the S_2_ generation (S_2G_ and S_2N_) were used for genetic marker analysis. As a control for the neoallotetraploid F_2_ population, diploid F_2_ populations were constructed from reciprocal crosses between diploid *M. guttatus* IM62 (IM-2x) and *M. nasutus* SF (IM-4x); and a single plant of each cross direction in the F_1_ generation was selfed to generate the F_2_ population (Figure 2A).

### Assessing evidence for homeologous recombination: Genetic marker analysis

Our first goal was to determine if homeologous recombination was occurring in neoallotetraploid *Mimulus* by using genetic marker data. DNA was extracted from 48 BC_1N_ individuals using a modified CTAB method (Kelly and Willis 1998). These 48 BC_1N_ individuals were genotyped at seven microsatellite loci known to differ between *M. guttatus* IM62 and *M. nasutus* SF. Each marker is located in the distal portion of its respective chromosome arm. In addition, 48 individuals, each from a separate S_2_ family (24 S_2G_ families and 24 S_2N_ families that were used to create the S_4_ families), were genotyped at 13 microsatellite loci, with one marker per linkage group. PCR conditions were as follows: 3 min at 95°, 12 cycles of 30 sec at 94°, 30 sec at 60°, and 45 sec at 72°, with annealing temperature reduced by 1° each cycle, followed by 32 cycles with annealing temperature held constant at 52°, and a final extension time of 20 min; the primer sequence for each marker can be found at http://www.mimulusevolution.org. S_2G_, S_2N_, and BC_1N_ progeny were then scored as either heterozygous or homozygous with the assistance of the GeneMarker software program (SoftGenetics LLC, State College, PA). To test for departures from models of chromosome pairing (Table 1), we constructed a 95% confidence interval based on the binomial distribution for each observed proportion of heterozygous individuals using the pooled marker data from each progeny class separately (S_2G_, S_2N_, BC_1N_). For the BC_1N_ pooled marker data set, we also conducted a *G*-test (log-likelihood ratio test) using Equation 17.4 as demonstrated in Box 17.1 of Sokal and Rohlf (1995), with the random chromosome model as our null hypothesis. For the six markers that had at least one homozygous individual, a heterogeneity *G*-test was conducted as per Box 17.4 of Sokal and Rohlf (1995).

To further assess the degree to which homologous chromosomes preferentially pair, we calculated the preferential pairing rate, *p*, which is a measure of how often homologous chromosomes pair with one another, rather than with their homeologous counterparts (Sybenga 1994). The preferential pairing rate (*p*) can vary from 0 to ⅔. At *p* = 0, each chromosome arm may pair randomly with any other homologous or homeologous chromosome arm, and chromosome arms are assumed to pair independently, allowing for quadrivalents to form. The preferential pairing rate reaches an upper bound at *p* = ⅔, which is derived from the fact that ⅓ of all bivalent pairings are between homologous chromosomes and ⅔ are between homeologous chromosomes. Thus, under a model of strict homologous pairing, homologous bivalents have a probability of 1, or ⅓ + *p*. Wu *et al.* (2001) developed a model for calculating the preferential pairing rate from genetic marker data rather than chromosome configurations at metaphase, as shown by Sybenga (1994); we used the model of Wu *et al.* (2001) to calculate the preferential pairing rate from the BC_1N_ progeny.

In the BC_1N_ progeny, there are three possible genotypes, GGNN, GNNN, and NNNN, with the SF-4x parent (NNNN) contributing NN gametes and the F_1_-4x (G_1_G_2_N_1_N_2_) parent contributing GG (G_1_G_1_, G_1_G_2_, G_2_G_2_), GN (G_1_N_1_, G_1_N_2_, G_2_N_1_, G_2_N_2_), or NN (N_1_N_1_, N_1_N_2_, N_2_N_2_) gametes. We directly solved for *p* using the genetic marker data and equations derived from Wu *et al.* (2001):

$$f\left(G_{1}G_{1}\right)+f\left(G_{2}G_{2}\right)+f\left(N_{1}N_{1}\right)+f\left(N_{2}N_{2}\right)=\alpha \left(2/3\text{-}3/2p^{2}\right),\;\mathrm{and}$$ (1)

f(G1N1)+f(G1N2)+f(G2N1)+f(G2N2)=2/9+1/3p+5/4p2+2/3(2/3-3/2p2-α(23-3/3p2)) (2) ,

where *p* = the preferential pairing factor, α is the double reduction rate, and *f*(G_i_G_j_), for example, is the observed or inferred frequency of a given gamete. Because our microsatellite data are not fully informative (*i.e.*, G_1_ = G_2_ and N_1_ = N_2_, and GGNN and GNNN genotypes are indistinguishable), we chose to make some simplifying assumptions that allowed us to calculate the preferential pairing rate directly from the data rather than to infer the missing genotypes using a expectation maximization algorithm, as suggested by Wu *et al.* (2001).

For each marker, we first assumed that an equal number of GG and NN gametes were created in the F_1_-4x parent, despite the fact that we were not able to infer the occurrence of GG gametes. Second, we assumed either that any NN or GG gametes derived from the F_1_ parent were the result of homeologous bivalent pairings or that any NN or GG gametes derived from the F_1_ parent were derived entirely from quadrivalent pairings, and a crossover occurred between the locus and the centromere, allowing for double reduction. In the case of the first scenario, equation 1 is set equal to zero, because G_1_G_1_, G_2_G_2_, N_1_N_1_, and N_2_N_2_ can only be derived from quadrivalent formation and subsequent double reduction. In the case of the second scenario, we assumed that three-eighths of all GG and NN gametes are derived from double reduction events; this frequency is expected if all nine possible quadrivalent arrangement and separation combinations (Figure S1) are considered. Because of our simplifying assumptions, we could not solve for the rate of double reduction (α). To evaluate whether the observed *p* fit the data better than a model with *p* = ⅔, likelihood ratio tests were conducted for each marker using the expected gamete frequencies and observed gamete counts, as described by Wu *et al.* (2001) and Curole and Hedgecock (2005).

### Common garden experiment and phenotypic analyses

Before the final common garden experiment in which all lines were measured, diploid and tetraploid parental (*M. guttatus* IM62 and *M. nasutus* SF), F_1_ and F_2_ lines, and two maternal lines for each of the 43 S_4G_ and 35 S_4N_ families, as well as seed from one maternal family from each of two *M. sookensis* populations (*M. sookensis* FAN and *M. sookensis* ROG from Vancouver Island, British Columbia, Canada, and Mehama, Oregon, respectively) were all generated simultaneously in the greenhouses at Duke University to standardize any environmental effects induced by the greenhouse over time. Neoallotetraploid F_1_ seed was freshly regenerated by recreating autotetraploid parental lines and reciprocally crossing them. Previously generated S_2G_ and S_2N_ seed was also grown in the final grow-out, but the seed was not matured at the same time in the greenhouse as all other seed. At the time of the final common garden experiment, and after the first generation of the F_1_ hybrid-derived neoallotetraploid lines (S_2_ generation), synthetic polyploid lines were assayed for DNA content as a proxy for ploidy to ensure that the unlikely event of a reversion to diploidy had not occurred. Flow cytometry was performed as described above and as by Modliszewski and Willis (2012).

For each plant grown and phenotyped, one to four seeds were placed onto well-moistened Fafard 4P soil-less potting medium in 2.5-inch pots. Flats were covered with humidity domes and placed at 4° with little ambient light for vernalization. All flats were removed from the cold room and placed in the greenhouse 6–8 days after sowing. After germination, pots were haphazardly randomized by moving pots from one flat to another, and extra seedlings were discarded, such that each pot contained one plant for the remainder of the experiment. Before the date of first flowering, flats were haphazardly moved about daily in the greenhouse to minimize spatial effects.

On the date of first flower, the first or second flower from each plant was phenotyped for the following traits, as described by Fishman *et al.* (2002): corolla width and length; corolla tube width and length; stamen length; and carpel (pistil) length. The width of the lower calyx was also measured for all plants using the distance between the two lower calyx lobes. Stigma-anther separation was calculated by measuring the difference in stamen and pistil length. Flowering time was measured from the date of germination to the date of first flowering. Pollen viability was assayed by macerating all four anthers from the first or second flower in a drop of lactophenol aniline blue (Kearns and Inouye 1993) placed on a microscope slide and then examining a haphazard selection of 100 pollen grains for viability under a light microscope at 100× magnification.

### Testing for an immediate effect of ploidy on phenotype: Analysis of means

To address whether polyploidization in *Mimulus* neoallotetraploids has immediate and direct phenotypic consequences, we calculated the mean value of all floral traits. Principal components analyses were first conducted using all phenotypic traits to visualize the distribution of F_1_ and F_2_ classes relative to parental and *M. sookensis* phenotypes. Principal component 1 (PC1) and principal component 2 (PC2) were subsequently included in all mean and variance calculations. To test if ploidy has a significant effect on phenotype, Tukey-Kramer HSD tests were performed among all classes (*e.g.*, IM-2x, IM-4x, F_1_-2x, F_1_-4x). We verified that comparison of means and variances by class (*e.g.*, F_1_-2x) was appropriate by testing for significant differences in the means within each reciprocal cross (*e.g.*, F_1G_-2x, F_1N_-2x) using a *t*-test. To test for normality, a Shapiro-Wilkes W test was conducted on each class for each trait. All statistical analyses of means and distributions were performed in JMP Pro 10.0 (SAS Institute, Inc., Cary, NC).

### Testing for an effect of ploidy on phenotype through homeologous recombination: Analyses of variance

In a typical diploid F_2_ population in which heritable genetic variation for a trait exists, an increase in variance, attributable to segregational variance, should be observed. To test for this increase in variance in the diploid and tetraploid F_2_ populations, F-tests, and Levene's test were conducted in R (R Development Core Team 2010); 95% confidence intervals for the coefficient of variation were calculated using equation 4.9 in Sokal and Rohlf (1995). To test for a significant family effect in the S_4G_ and S_4N_ families, a nested ANOVA was conducted separately for each trait and also for PC1 and PC2, with both family and maternal lines (nested within family) as random effects, using the REML computation in JMP Pro 10.0 (SAS Institute, Inc.). Broad-sense heritabilities (*H^2^*) based on the diploid and tetraploid F_2_ populations were also calculated for each trait as *H^2^* = V_G_/V_P_, where V_G_ = Var(F_2_)−V_E_, and V_E_ was calculated from parental and F_1_ variance.

## Results

### Identification of synthetic tetraploid lineages

From the F_1G_-2x (*M. guttatus* × *M. nasutus*) and F_1N_-2x (*M. nasutus* × *M. guttatus*) seed treated with 0.01% colchicine, one F_1G_-4x and one F_1N_-4x tetraploid individual was found after screening 59 and 137 individuals, respectively; these two plants were used to generate the S_4_ families. No tetraploid *M. guttatus* IM62 or *M. nasutus* SF was found among the 81 and 27 individuals (respectively) screened that were treated with 0.0001%–0.01% colchicine. In treatments with 0.05% and 0.1% colchicine, 11 neoautotetraploid lines of *M. guttatus* IM62 (IM-4x) and two neoautotetraploid *M. nasutus* SF (SF-4x) were identified after screening 44 and 337 individuals, respectively. Interestingly, although the rate of conversion to tetraploidy was quite low in *M. nasutus* compared to *M. guttatus*, the two tetraploid *M. nasutus* individuals were the result of the 0.05% colchicine treatment. Likewise, more *M. guttatus* IM62 individuals (seven *vs.* four) were converted to tetraploids at the 0.05% treatment level. We attempted to convert other lines of *M. guttatus* and *M. nasutus* to tetraploids but often had limited success with *M. nasutus* and had varying success with *M. guttatus* (data not shown), suggesting that there may be a genetic component to the observed variation. Although all individuals were screened regardless of appearance, by casual observation the leaves of neotetraploids induced via colchicine tended to have a thicker and darker appearance, and pollen grains were larger. Flow cytometric analysis of neoautotetraploid lines revealed that the autopolyploids had approximately double the DNA content of their diploid progenitors, as did neoallotetraploid F_1_ individuals (2.04 ± 0.018; n = 7) (Table S1). A small but significant decrease in 2C DNA content was observed in the neoallotetraploid F_2_ population (2.00 ± 0.007; n = 16) when compared to the F_1_-4x class (Table S1).

### Genetic marker analysis reveals limited evidence for homeologous recombination

Depending on the model of chromosome pairing, the expected genotypic frequencies in the S_2_ and BC_1N_ progeny will vary (Table 1). The progeny examined can be classified into two groups: heterozygous (GGNN, GGGN, and GNNN genotypes, with “G” indicating the *M. guttatus* allele and “N” indicating the *M. nasutus* allele) and homozygous (GGGG and NNNN). In the S_2_ progeny, all five genotypes can occur, but homeologous pairing can only be confirmed using our genetic marker data if a quadruple homozygote is formed. In BC_1N_ progeny, only GGNN, GNNN, and NNNN individuals can be formed, regardless of the chromosome pairing model. BC_1N_ progeny scored as heterozygous are either GGNN or GNNN; although GGNN genotypes are the result of homeologous pairing, they cannot be detected using the microsatellite data. Under even the most modest of homeolog pairing models, one-sixth of BC_1N_ progeny are expected to be quadruple homozygotes that have lost the *M. guttatus* allele (Table 1).

The genetic marker data revealed that homeologous recombination is occurring at an exceedingly low, and in some cases nonexistent, rate. In the S_2_ progeny, no instances of marker loss were observed at any of the 13 loci. The probability of failing to observe a quadruple homozygote as calculated by (5/6)^24^ was 25% under the least stringent random chromosome model, and the probabilities of failing to observe a quadruple homozygote for the total number of individuals observed over all markers in the S_2G_ and S_2N_ classes are 2.14×10^−8^ and 2.26×10^−8^, respectively. For the combined marker data at S_2G_ and S_2N_ classes, the 95% confidence interval under a binomial distribution incorporates only the homolog pairing model. On a per-marker basis, no chromosome pairing model can be excluded for either S_2_ class (Table 2).

**Table 2:** Results of marker screening in S_2_ and BC_1N_ individuals

**Class**	**No. Markers or Marker ID**	**Marker Location**	**Total No. Heterozygous Individuals**	**P(fail), Random Chromosome**	**No. Individuals With Marker Loss***^a^*	**Apparent Heterozygosity**	**Exact Binomial Test Confidence Interval**	**Preferential Pairing Factor***^b^*	***G*-test**
S_2G_	13	NA	309	2.14E−08	0	1.00	0.99–1.00	NA	—
S_2N_	13	NA	308	2.26E−08	0	1.00	0.99–1.00	NA	—
S_2G_/S_2N_	1	NA	23 or 24	0.25 or 0.27	0	1.00	0.85–1.00	NA	—
BC_1N_	MgSTS_98	1, Left arm	46	NA	2 (1)	0.96 (0.98)	0.857–0.995	0.54‡	—
	MgSTS_787	2, Left arm	47	NA	1 (0)	0.98 (1.00)	0.889–0.999	0.60^NS^	—
	MgSTS_724	6, Left arm	45	NA	3 (2)	0.93 (0.96)	0.828–0.987	0.46†	—
	MgSTS_376^	7, Left arm	48	1.58E−04	0	1.0000	0.926–1.000	0.67^NA^	—
	MgSTS_358^	11, Right arm	47	NA	1 (0)	0.98 (1.00)	0.889–0.999	0.60^NS^	—
	MgSTS_780	11, Left arm	47	NA	1 (0)	0.98 (1.00)	0.889–0.999	0.60^NS^	—
	MgSTS_847	1, Left arm	46	NA	2 (1)	0.96 (0.98)	0.857–0.995	0.54‡	—
	7	NA	336	2.48E−27	10 (4)	0.97 (0.99)	0.946–0.986	NA	9.07E−16

Marker location is indicated by chromosome number, which corresponds to linkage group numbers. A caret (^) is used to indicate markers that were analyzed in both the S_2_ and BC_1N_ data sets. P(fail), Random Chromosome indicates the probability of failing to observe a quadruple homozygote (*i.e.*, NNNN or GGGG) under the random chromosome model. The preferential pairing factor (*p*) was calculated using the number of individuals with marker loss for the full data set, including the single individual with six instances of marker loss. For the replicated *G*-test, the null hypothesis was that chromosomes abided by the random chromosome assortment model in Table 1. NS, not significant; NA, not available (did not calculate).

a The values in parentheses indicate the number of individuals after exclusion of the single BC_1N_ individuals with six instances of marker loss.

b Significance of p-values are coded as follows: *<0.001; †<0.01; ‡<0.05.

In the BC_1N_ progeny, 10 instances of marker loss were observed over the seven markers surveyed, with one BC_1N_ individual contributing to six of the 10 observed instances of marker loss. Fifty-six instances of marker loss (one-sixth of the 336 marker/progeny combination) were expected to occur under the most conservative model of homeologous chromosome pairing. The 95% confidence interval of the proportion of apparent heterozygosity, as calculated using an exact binomial test for the entire set of marker data, is 0.946–0.986. A *G*-test of the entire BC_1N_ data set under the random chromosome model resulted in a rejection of the null hypothesis (p-value = 9.07×10^−6^), indicating that the data do not fit the least stringent model of nonhomologous chromosome pairing; when pooled, the markers did not show significant heterogeneity (G_H_ = 1.99, p-value = 0.85). When each marker in the BC_1N_ data set was analyzed separately, only one marker (MgSTS_727) had a confidence level overlapping the least stringent nonhomologous chromosome pairing model. Overall, the genetic marker data did not display a pattern of inheritance strictly congruent with any of the models of homeologous chromosome pairing.

To provide further insight into the nature of chromosome pairing in synthetic allotetraploid *Mimulus*, we calculated the preferential pairing rate (*p*) (Sybenga 1994) from the BC_1N_ progeny. If chromosome pairings are exclusively homologous, then *p* = ⅔. Our genetic marker data indicate that *p* ranges from 0.46 to 0.67 (Table 2). Assuming either 100% bivalent pairings or 100% quadrivalent pairings has little effect on *p* (data not shown), and thus the values reported here were all calculated under the assumption of 100% bivalent pairings, which actually results in a slightly smaller value for *p*.

### Analysis of means: Polyploidization results in large-flowered *Mimulus*

**Figure 3:**
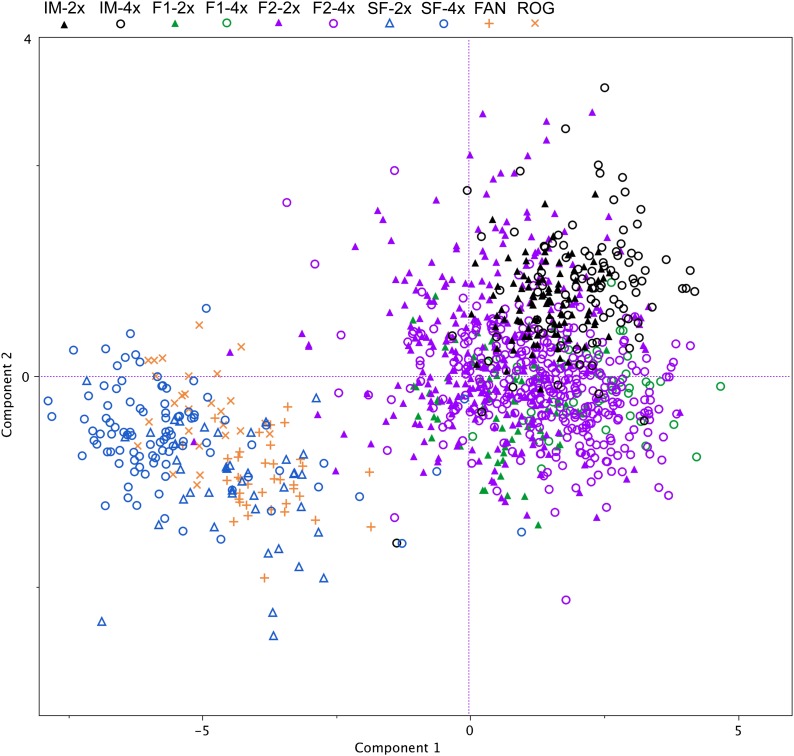
PCA scores for principal component 1 (PC1) and principal component 2 (PC2). Symbols are coded by shape as follows: ▴ and ▵ (diploids); ○ (synthetic tetraploids); orange x (*M. sookensis* ROG); and orange + (*M. sookensis* FAN). Symbols are color-coded to indicate *M. nasutus* SF (blue), *M. guttatus* IM62 (black) F_1_ hybrids (light green), and F_2_ hybrids (purple).

**Figure 4:**
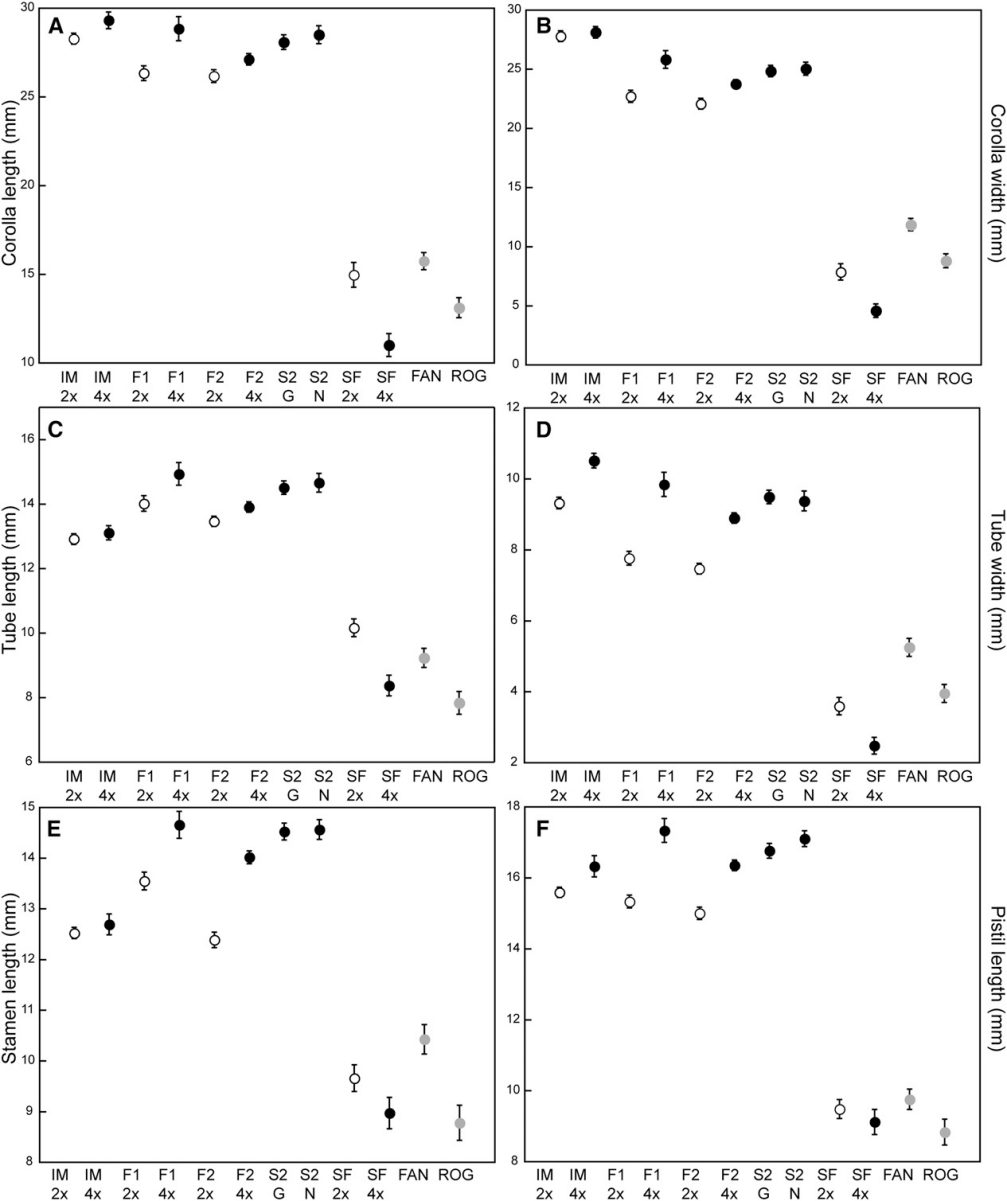

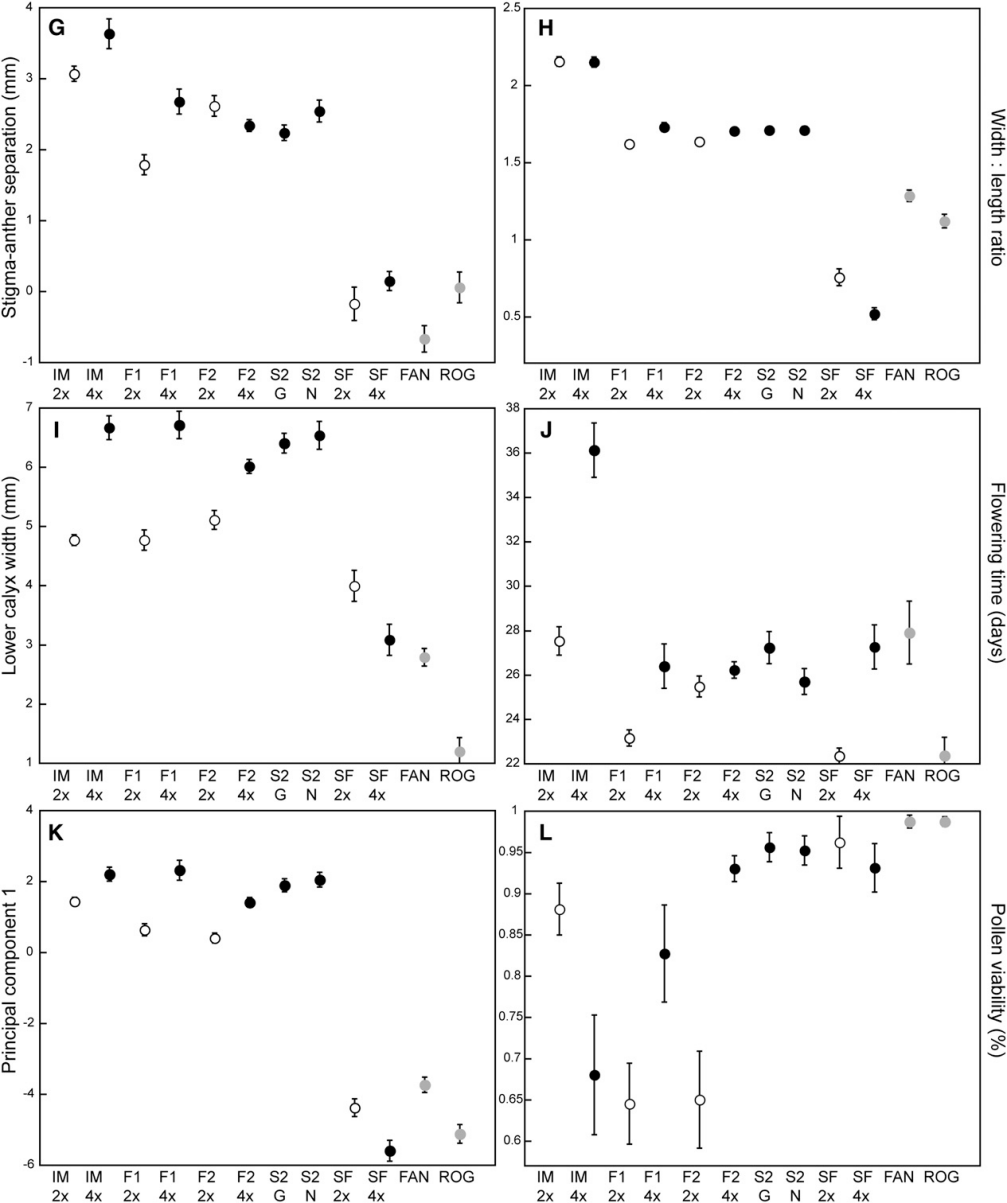
Means and 95% confidence intervals for floral traits. IM, *M. guttatus* IM62; SF, *M. nasutus* SF; FAN, *M. sookensis* FAN; and ROG, *M. sookensis* ROG. Ploidy for parental, for F_1_, and for F_2_ populations are indicated with 2x (diploid) or 4x (neotetraploid) as appropriate; both S_2_ classes are tetraploid. Means are shaded to indicate ploidy as follows: diploids (white), artificial neotetraploids (black), and naturally occurring allotetraploids (gray).

To determine the effects of polyploidization on floral phenotype in *Mimulus*, floral traits were measured in neoautotetraploids and neoallotetraploid F_1_ and F_2_ hybrids and compared to diploid parental and hybrid lines. Phenotypic analysis revealed that F_1_ and F_2_ neoallotetraploid *Mimulus* are large-flowered (Figure 3 and Figure 4). Principal components analysis revealed that floral traits are highly correlated, and the first two principal components (PC1 and PC2) capture 77.21% and 10.02% of the phenotypic variation, respectively (Table S2), with most floral traits positively loaded on the first principal component. Tests for departures from normality revealed that most traits did not fit a normal distribution and that a log-normal distribution also did not fit the data, with the exception of width:length ratio (Table S3). Subsequently, with the exception of width:length ratio, no transformations were performed. The non-normality of the data is not expected to have an effect on our tests for differences of the means.

Our measurement of floral traits reveals three distinct patterns (Figure 3, Figure 4, and Table S4). First, the two measured *M. sookensis* lines cluster significantly with diploid and neoautotetraploid *M. nasutus*, although they are statistically different from each other and *M. nasutus* for some traits. Second, dominance of *M. guttatus* floral characters is upheld in the neoallotetraploid F_2_ plants and was also observed in diploid hybrids (Figure 4 and Table S4) (Fishman *et al.* 2002). Third, in *M. guttatus* and F_1_ hybrids, ploidy appears to cause an increase in flower size, although the difference is not always significant, whereas autotetraploid *M. nasutus* have smaller flowers than diploid *M. nasutus*. Two other patterns were observed from the phenotypic data. First, whereas overall there was little variation in flowering time (as measured by days from germination to date of first flower), the neoautotetraploid lines of *M. guttatus* (IM-4x) (Table S4 and Figure 4) flowered more than 8 days later than all other lines. We observed that these individuals lingered as seedlings with only cotyledons for an extended period of time before growing true leaves and bolting. Last, in the neoautotetraploid lines, ploidy appears to cause a decrease in pollen viability, although the difference was not statistically significant in the *M. nasutus* lines (Figure 4 and Table S4). In contrast, the neoallotetraploid lines had significantly higher pollen viability than did their diploid counterparts; pollen viability in neoallotetraploid lines was equivalent to pollen viability in diploid *M. nasutus* and *M. sookensis*, which had the highest pollen viability among all classes.

### Analysis of variance: Homeologous recombination does not significantly contribute to phenotypic variation

To determine if the low observed levels of homeologous recombination observed in genetic marker data from BC_1N_ progeny and S_2_ families could result in phenotypic variation with an underlying heritable genetic basis, variance components of neoallotetraploid F_2_s and S_4_ families were calculated. In the diploid F_2_ population, a significant increase in variation was observed at 10 of 11 traits, according to Levene's test, which is robust to asymmetry of the mean, with the variation seen in diploid F_2_s for tube length being of marginal significance (Table 3). In contrast, only one trait out of 11 (PC2) showed significantly elevated levels of variation in the neoallotetraploid F_2_ population after accounting for traits in which subdividing the F_2_ class was appropriate (Table 3 and Table S5; tube length, corolla width, corolla length, lower calyx width, and PC1).

**Table 3:** Variance, broad-sense heritability (*H*^2^), and results of tests for homogeneity of variance between F_1_ and F_2_ classes

				**F_1_-2x**	**F_2_-2x**	**F_1_-2x**	**F_2_-2x**			**F_1_-4x**	**F_2_-4x**	**F_1_-4x**	**F_2_-4x**
**Trait**		**F_1_-2x**	**F_2_-2x**	***guttatus***		***nasutus***		**F_1_-4x**	**F_2_-4x**	***guttatus***		***nasutus***	
	Variance	3.000	17.080	5.304	15.433	0.875	18.838	13.240	12.240	15.748	13.900	7.103	9.722
Flowering time	Significance	*/*/‡		*/‡		*/†		NS/NS/NS		NS/NS		NS/NS	
(FT)	*H*^2^	0.72		0.62		0.80		*−0.87*		—		—	
	Variance	0.831	1.803	1.081	1.799	0.605	1.802	1.546	1.914	1.868	2.212	1.239	1.616
Tube width	Significance	*/†/‡		§/NS		*/*		NS/NS/NS		NS/NS		NS/NS	
(TW)	*H*^2^	0.53		—		0.60		*0.27*		—		—	
	Variance	1.311	1.973	1.359	1.791	1.017	2.145	1.640	2.386	2.002	2.404	1.252	2.139
Tube length	Significance	‡/§/‡		NS/NS		†/‡		NS/NS/NS		NS/NS		§/§	
(TL)	*H*^2^	0.41		—		0.53		*0.25*		—		—	
	Variance	5.994	15.949	7.682	15.432	4.358	16.455	7.598	11.851	9.462	13.338	6.520	8.831
Corolla width	Significance	*/*/‡		‡/‡		*/†		§/NS/‡		NS/NS		NS/NS	
(CW)	*H*^2^	0.61		0.54		0.67		*0.37*		—		—	
	Variance	3.883	10.249	4.523	8.322	2.938	12.191	6.084	8.940	6.615	9.694	5.870	7.301
Corolla length	Significance	*/*/‡		‡/‡		*/†		§/NS/NS		NS/NS		NS/NS	
(CL)	*H*^2^	0.54		0.40		0.66		*0.20*		—		—	
	Variance	0.687	1.826	0.873	1.988	0.484	1.420	0.920	1.408	1.204	1.631	0.700	1.089
Stamen length	Significance	*/*/‡		†/†		*/‡		§/NS/NS		NS/NS		NS/NS	
(SL)	*H*^2^	0.61		0.59		0.56		*0.04*		—		—	
	Variance	0.720	2.435	0.803	2.553	0.643	2.327	1.474	1.949	1.707	2.123	1.238	1.730
Pistil length	Significance	*/*/‡		*/*		*/†		NS/NS/NS		NS/NS		NS/NS	
(PL)	*H*^2^	0.67		0.67		0.67		*0.08*		—		—	
Stigma-anther	Variance	0.451	1.670	0.473	1.884	0.433	1.170	0.414	0.622	0.371	0.760	0.444	0.475
separation	Significance	*/*/‡		*/*		*/‡		§/NS/‡		§/NS		NS/NS	
(SAS)	*H*^2^	0.68		0.71		0.55		*0.02*		—		—	
Corolla width:	Variance	0.004	0.019	0.007	0.018	0.002	0.020	0.004	0.007	0.002	0.010	0.005	0.004
length ratio	Significance	*/*		†/†		*/*		†/§/‡		*/‡		NS/NS	
(WLR)	*H*^2^	0.49		0.42		0.54		*−0.16*		0.22		—	
Lower	Variance	0.570	1.854	0.327	1.900	0.670	1.810	0.713	1.224	0.647	1.204	0.782	1.059
calyx width	Significance	*/*/‡		*/†		*/†		‡/†/‡		NS/§		NS/§	
(LXW)	*H*^2^	0.71		0.78		0.68		0.12		—		—	
Principal	Variance	0.644	1.596	0.758	1.546	0.481	1.657	1.039	1.596	1.268	1.724	0.872	1.334
component 1	Significance	*/†/‡		‡/‡		*/†		§/§/‡		NS/NS		NS/NS	
(PC1)	*H*^2^	0.57		0.52		0.64		*0.16*		—		—	
Principal	Variance	0.302	0.901	0.344	0.997	0.245	0.617	0.177	0.386	0.198	0.472	0.168	0.274
component 2	Significance	*/*/NA		*/*		*/‡		†/‡/NA		‡/NS		NS/NS	
(PC2)	*H*^2^	0.62		0.63		0.48		0.22		0.34		—	

For the tests of homogeneity of variance, the results of three tests are reported for the nonsubdivided F_1_ and F_2_ classes (F-test/Levene's test/CV*), whereas the results of two tests (F-test/Levene's test) are reported for the subdivided categories (*e.g.*, F_1G_-2x *vs.* F_2G_-2x). Significance of p-values are coded as follows: *<0.001; †<0.01; ‡<0.05; §>0.05 and <0.10. For heritability calculations, — or italic font is used to indicate that the calculation of heritability is not appropriate. For PC2, calculations of the CV are not appropriate, because the means for some of the classes are negative; this is indicated with NA. Trait abbreviations are given in parentheses. NS, not significant; NA, not appropriate.

Broad-sense heritability of floral traits and flowering time calculated from the diploid F_2_ cross ranged from 0.41 for tube length to 0.72 for flowering time. In contrast, the heritabilities for floral traits and flowering time ranged from −0.87 for flowering time to 0.37 for corolla width in the tetraploid cross. Negative heritabilities indicate an obvious lack of heritable genetic variation, and heritability of traits that do not demonstrate segregational variance is not meaningful. For PC2, the only trait to display a significant level of segregating variation in the tetraploid F_2_ cross, the broad-sense heritability was 0.22, whereas it was 0.62 in the diploid F_2_ cross (Table 3).

For the S_4_ families, we tested the hypothesis that individuals from a family were more phenotypically similar to each other than they were to a random individual, a relationship that should arise if heritable genetic variation exists for a given trait. For the S_4_ families derived from S_1_ tetraploids with *M. guttatus* as the maternal parent, a significant effect of family was found for six of 11 traits (Table 4). For all six traits, the lower bound of the 95% confidence limit was very close to zero. In contrast, for the S_4_ families derived from S_1_ tetraploids with *M. nasutus* as the maternal parent, a significant effect of family was not found in any of the 11 traits examined (Table 4). For lower calyx width and stamen length, a significant maternal effect was found in the S_4N_ families, but no significant maternal effects were found in the S_4G_ families for any trait. Maternal effects were measured by splitting the S_4_ families into subfamilies in the penultimate generation and measuring three individuals from each subfamily during the common garden experiment.

**Table 4:** Variance components, SE, significance at α = 0.05, and percent total variance for S_4_ families calculated separately based on original synthetic tetraploid F_1_

	**S_4G_**				**S_4N_**			
**Trait**	**Random Effect^a^**	**Variance ± SE**	**Significance**	**% Total**	**Random Effect**	**Variance ± SE**	**Significance**	**% Total**
**FT**	Family	10.64 ± 5.00	‡	22.35	Family	2.11 ± 2.45	NS	7.08
	Maternal (family)	5.53 ± 4.45	NS	11.61	Maternal (family)	4.16 ± 3.12	NS	13.94
	Residual	31.45 ± 4.08	‡	66.04	Residual	23.56 ± 2.93	‡	78.99
	Total	47.61 ± 5.53		100.00	Total	29.83 ± 3.14		100.00
**TW**	Family	0.26 ± 0.16	NS	12.68	Family	0.21 ± 0.13	NS	13.41
	Maternal (family)	−0.04 ± 0.18	NS	0.00	Maternal (family)	0.10 ± 0.14	NS	6.13
	Residual	1.82 ± 0.22	‡	87.32	Residual	1.25 ± 0.16	‡	80.47
	Total	2.09 ± 0.28		100.00	Total	1.56 ± 0.17		100.00
**TL**	Family	0.47 ± 0.27	NS	13.87	Family	0.19 ± 0.23	NS	7.59
	Maternal (family)	0.03 ± 0.29	NS	0.96	Maternal (family)	0.42 ± 0.29	NS	16.57
	Residual	2.88 ± 0.36	‡	85.18	Residual	1.91 ± 0.24	‡	75.84
	Total	3.38 ± 0.35		100.00	Total	2.52 ± 0.27		100.00
**CW**	Family	2.62 ± 1.18	‡	18.39	Family	0.98 ± 0.92	NS	9.46
	Maternal (family)	−0.34 ± 1.06	NS	0.00	Maternal (family)	1.44 ± 1.08	NS	13.86
	Residual	11.61 ± 1.42	‡	81.61	Residual	7.98 ± 0.99	‡	76.69
	Total	14.22 ± 1.85		100.00	Total	10.41 ± 1.11		100.00
**CL**	Family	2.66 ± 1.09	‡	20.01	Family	0.82 ± 0.87	NS	9.07
	Maternal (family)	−0.43 ± 0.94	NS	0.00	Maternal (family)	1.74 ± 1.03	NS	19.30
	Residual	10.65 ± 1.31	‡	79.99	Residual	6.45 ± 0.80	‡	71.64
	Total	13.31 ± 1.70		100.00	Total	9.00 ± 0.98		100.00
**SL**	Family	0.36 ± 0.17	‡	16.19	Family	−0.02 ± 1.12	NS	0.00
	Maternal (family)	−0.10 ± 0.17	NS	0.00	Maternal (family)	0.37 ± 0.17	‡	28.81
	Residual	1.87 ± 0.23	‡	83.81	Residual	0.91 ± 0.11	‡	71.19
	Total	2.23 ± 0.29		100.00	Total	1.28 ± 0.18		100.00
**PL**	Family	0.65 ± 0.29	‡	18.41	Family	0.19 ± 0.22	NS	8.73
	Maternal (family)	−0.09 ± 0.28	NS	0.00	Maternal (family)	0.50 ± 0.27	NS	22.77
	Residual	2.89 ± 0.36	‡	81.59	Residual	1.52 ± 0.19	‡	68.50
	Total	3.55 ± 0.47		100.00	Total	2.21 ± 0.25		100.00
**SAS**	Family	0.06 ± 0.07	NS		Family	0.09 ± 0.16	NS	3.72
	Maternal (family)	0.11 ± 0.08	NS	13.82	Maternal (family)	−0.19 ± 0.20	NS	0.00
	Residual	0.64 ± 0.08	‡	78.61	Residual	2.37 ± 0.29	‡	96.28
	Total	0.81 ± 0.08		100.00	Total	2.46 ± 0.33		100.00
**WLR**	Family	0.00 ± 0.00	NS	15.34	Family	0.00 ± 0.00	NS	13.05
	Maternal (family)	0.00 ± 0.00	NS	3.74	Maternal (family)	0.00 ± 0.00	NS	0.89
	Residual	0.01 ± 0.00	‡	80.93	Residual	0.01 ± 0.00	‡	86.06
	Total	0.01 ± 0.00		100.00	Total	0.01 ± 0.00		100.00
**LCW**	Family	0.32 ± 0.16	NS	17.74	Family	0.11 ± 0.16	NS	7.31
	Maternal (family)	0.18 ± 0.16	NS	10.36	Maternal (family)	0.45 ± 0.20	‡	29.31
	Residual	1.28 ± 0.16	‡	71.90	Residual	0.98 ± 0.12	‡	63.38
	Total	1.78 ± 0.19		100.00	Total	1.55 ± 0.17		100.00
**PC1**	Family	0.42 ± 0.20	‡	17.77	Family	0.13 ± 0.15	NS	7.76
	Maternal (family)	−0.02 ± 0.19	NS	0.00	Maternal (family)	0.30 ± 0.19	NS	18.64
	Residual	1.95 ± 0.24	‡	82.23	Residual	1.20 ± 0.15	‡	73.60
	Total	2.37 ± 0.31		100.00	Total	1.63 ± 0.18		100.00
**PC2**	Family	0.02 ± 0.03	NS	4.09	Family	0.02 ± 0.08	NS	1.63
	Maternal (family)	0.06 ± 0.04	NS	15.81	Maternal (family)	−0.05 ± 0.11	NS	0.00
	Residual	0.33 ± 0.04	‡	80.10	Residual	1.13 ± 0.14	‡	98.37
	Total	0.41 ± 0.04		100.00	Total	1.15 ± 0.16		100.00

S_4G_ refers to the S_4_ family data derived from synthetic F_1_ with *M. guttatus* as the maternal parent, whereas S_4N_ refers to the refers to the S_4_ family data derived from synthetic F_1_ with *M. nasutus* as the maternal parent. Family and maternal parent within family were treated as random effects, with maternal parent nested within family. Significance of p-values are coded as follows: *<0.001; †<0.01; ‡<0.05. FT, flowering time; TW, tube width; TL, tube length; CW, corolla width; CL, corolla length; WLR, tube width:corolla length ratio; SL, stamen length; PL, carpel (pistil) length; SAS, stigma-anther separation; LXW, lower calyx width; PC1, principal component 1; PC2, principal component 2; NS, not significant.

## Discussion

The objective of this study was to assess the effects of polyploidization on genetic and phenotypic variation in *Mimulus*. Using synthetic neoallotetraploid *Mimulus* derived from *M. guttatus* and *M. nasutus*, we report three key findings. First, we used genetic marker data to show that homeologous pairing and recombination are almost negligible in neoallotetraploid *Mimulus*. We then showed that polyploidization results in large-flowered neoallotetraploid *Mimulus*, in contrast to naturally occurring *M. sookensis*. Finally, we demonstrated that the low levels of homeologous recombination do not contribute to significant levels of phenotypic variation, suggesting that polyploidization *per se* is not a major driver of genetic and phenotypic variation in neoallotetraploid *Mimulus*.

Analysis of genetic marker data led us to reject models of homeologous chromosome pairing. Although some fragment loss was observed in BC_1N_ progeny, the level of fragment loss observed (10 instances) was far below the level expected (56 instances) if homeologous chromosomes regularly pair, and we observed no instances of marker loss in the S_2_ progeny. In addition, the preferential pairing rate was found to be significantly different from a model in which *p* = ⅔ (*i.e.*, strict homologous pairings) in three of the seven loci examined in the BC_1N_ progeny. The binomial tests, replicated *G*-test, and preferential pairing rate all suggest that although chromosomes may often preferentially pair with their homologous counterpart, it is not a strict association. In the genetic marker data presented here, we cannot distinguish between rare homeologous recombination events causing the loss of one parental genome fragment with replacement from the other fragment, and deletion of one fragment without replacement. It is thus possible that the 10 instances of observed fragment loss are attributable to fragment deletion caused possibly by ectopic or unequal recombination rather than homeologous recombination. Nevertheless, it is still possible to test if fragment loss, regardless of the mechanism, generates a measurable phenotypic effect.

### Polyploidization results in large-flowered neoallotetraploid *Mimulus*

Polyploidization can result in noticeable phenotypic effects, as first observed in the tetraploid “*gigas*” mutants of *Oenothera lamackiana* (DeVries 1915 and references therein). We found that neoallotetraploid *Mimulus* are large-flowered and do not resemble naturally occurring *M. sookensis*. The large flower size of neoallotetraploid F_1_ and F_2_ plants suggests that the dominance relationships of *M. guttatus* and *M. nasutus* floral loci remain unchanged after polyploidization. Interestingly, there also appears to be a dosage effect of floral loci; neoautotetraploid *M. guttatus*, which contains four copies of each of the many loci contributing to large flower size, is larger than diploid *M. guttatus*, whereas neoautotetraploid *M. nasutus*, which contains four rather than two small-flower alleles at each of the loci contributing to flower size, is smaller than diploid *M. nasutus*. Unlike in *Arabidopsis* (Madlung *et al.* 2005), induction of polyploidy did not result in phenotypic instability.

It is unlikely that the use of different lines of *M. guttatus* and *M. nasutus* would result in small-flowered neoallotetraploid *Mimulus*. For this to occur, there would have to be alternative flower size alleles in *M. guttatus* and *M. nasutus* that interact to create small-flowered F_1_ hybrids. Range-wide crossing experiments between eight pairs of *M. guttatus* and *M. nasutus* lines always resulted in intermediate to large-flowered F_1_ hybrids (Martin and Willis 2010; N.H. Martin, personal communication), suggesting that differential epistatic interactions between flower size alleles derived from different populations of *M. guttatus* and *M. nasutus* could not combine to create small-flowered neoallotetraploids. It is also unlikely that a small-flowered *M. guttatus* was the progenitor of *M. sookensis*. Because *M. sookensis* has recurrently formed throughout its range (Modliszewski and Willis 2012), a small-flowered *M. guttatus* would have to form an unlikely mating with small-flowered *M. nasutus*; it would also have to be present at a fairly high frequency or produce a disproportionate amount of unreduced gametes. Given that the larger flower size of *M. guttatus* is likely to be the ancestral state of the *M. guttatus* and *M. nasutus* based on phylogenetic relationships within *Mimulus* (Beardsley *et al.* 2004), and the fact that no small-flowered *M. guttatus* was observed, this scenario is highly unlikely. These observations, together with our data, suggest that polyploidization of *M. guttatus* and *M. nasutus* hybrids will result in large neoallotetraploids regardless of the *M. guttatus* and *M. nasutus* lines used, and that *M. sookensis* was derived from a large-flowered *M. guttatus*.

### Low levels of homeologous recombination do not significantly enhance phenotypic variation in neoallotetraploid *Mimulus*

In the segregating diploid F_2_ population, all traits exhibited evidence of segregational variance and had significant levels of broad-sense heritability, as is expected with segregation and independent assortment. Analyses of variance in tetraploid F_2_s and S_4_ families suggests that fragment loss (if occurring) is not likely to create substantial genetic variation that has a corresponding phenotypic effect. In support of this conclusion, broad-sense heritability was much lower in the tetraploids relative to diploids. Although broad-sense heritability is expected to be lower in tetraploids relative to diploids with similar phenotypic ranges because of the expected decreased variance in tetraploids, in our study the parental neoautotetraploid lines have a larger phenotypic range than diploid parental lines, which should increase the expected genetic variance in F_2_s and thus should increase the estimate of broad-sense heritability.

An additional line of indirect evidence is suggestive of infrequent homeologous pairing in neoallotetraploid *Mimulus*. The high levels of pollen viability observed in neoallotetraploids, compared to the decreased levels of pollen viability in neoautotetraploids (Table S4 and Figure 4.), suggest that chromosomes are regularly pairing in bivalents in the neoallotetraploids, but in quadrivalents in the neoautotetraploids. However, it may be that further assessment of pollen viability or germinability may reveal that the difference in viability of pollen between neoautotetraploids and neoallotetraploids is not as great as originally observed. A negative correlation between quadrivalent formation and fertility has been previously observed in wheat and other species (Ozkan *et al.* 2001; Ramsey and Schemske 2002).

### Implications for phenotypic evolution in polyploids

Overall, the nuances of the data suggest that there is not rampant pairing of homeologous chromosomes, but that occasionally homeologous chromosomes may pair or fragment loss through another deletion mechanism may be occurring. Low to nonexistent levels of homeologous recombination will slow the process of phenotypic evolution, unlike the evolution of flowering time observed in *Brassica napus* (Schranz and Osborn 2000, 2004). In *Gossypium* allotetraploids, although homeologous recombination has been confirmed, the timing of homeologous recombination events is scattered over the evolutionary history of cotton (Salmon *et al.* 2010). A similar phenomenon may be occurring in *M. sookensis*. Alternate pathways of formation, such as through an outcrossing autotetraploid progenitor rather than a diploid F_1_ hybrid, may contribute to an initial augmentation of genetic variation, but if the polyploid lineage is formed from only a few individuals, then homologous pairing will still limit genetic variation in the allopolyploids. At present, it appears that the best approach to determining the genetic mechanisms responsible for the evolution of flower size in *M. sookensis* is to examine naturally occurring *M. sookensis* on a genome-wide level for evidence of homeologous recombination or to directly address the genetic basis of flower size via QTL mapping.

Although polyploidization itself may be widespread among plants (Masterson 1994; Otto and Whitton 2000; Blanc and Wolfe 2004; Cui *et al.* 2006), the effects of polyploidization as observed in any single polyploid system do not appear to be universally applicable. For example, the rapid fragment loss and cytosine methylation observed in *Aegilops-Triticum* wheat (Ozkan *et al.* 2001; Shaked *et al.* 2001) do not occur in *Gossypium*, which instead demonstrates biased expression dominance on a genome-wide level (Liu *et al.* 2001; Flagel *et al.* 2008; Chaudhary *et al.* 2009; Rapp *et al.* 2009). Likewise, rapid genomic changes have not been documented in *Spartina anglica* (Baumel *et al.* 2002; Ainouche *et al.* 2004; Salmon *et al.* 2005). In artificial *Arabidopsis suecica*, ∼11% of the genes exhibit a departure from expression additivity, but the phenotype of early generation synthetic *A. suecica* is quite similar to natural *A. suecica* (Wang *et al.* 2004), whereas the effects of polyploidization on gene expression differ in autotetraploid *Arabidopsis* lines (Yu *et al.* 2010). Additionally, early-generation synthetic allotetraploid *Nicotiana tabacum* display evidence of loss of 25%–60% of the repetitive fragments analyzed but appear phenotypically similar to domesticated *N. tabacum* (Skalická *et al.* 2005). Thus, it is not clear if fragment loss and expression nonadditivity observed in synthetic allotetraploids result in genetic variation that has a high (and observable) impact on phenotypic evolution. The findings we present here in *Mimulus* suggest yet another variation on a theme in polyploid evolution. One cannot assume that homeologous recombination or fragment loss will occur and allow for rapid recapitulation of the phenotype observed in natural or crop allopolyploids. Rather, the trajectory of phenotypic evolution may have been slow in the allotetraploid *M. sookensis* and perhaps also in other polyploid systems.

## Supplementary Material

Supporting Information
