# Supplementary material for: Near-Absent Levels of Segregational Variation Suggest Limited Opportunities for the Introduction of Genetic Variation Via Homeologous Chromosome Pairing in Synthetic Neoallotetraploid Mimulus
Source: G3 (Bethesda). 2014 Jan 27;4(3):509–22. doi: 10.1534/g3.113.008441 (PMC3962489; doi:10.1534/g3.113.008441)
Supplement: Supporting Information [file supp_4_3_509__index.html]

Near-Absent Levels of Segregational Variation Suggest Limited Opportunities for the Introduction of Genetic Variation Via Homeologous Chromosome Pairing in Synthetic Neoallotetraploid Mimulus — Supporting Information 

# Near-Absent Levels of Segregational Variation Suggest Limited Opportunities for the Introduction of Genetic Variation Via Homeologous Chromosome Pairing in Synthetic Neoallotetraploid *Mimulus*

## Supporting Information for Modliszewski and Willis, 2014

**Files in this Data Supplement:**

- Supporting Information - Figure S1, File S1, and Tables S1-S5 (PDF, 735 KB)
- Figure S1 - Quadrivalent arrangements (PDF, 339 KB)
- File S1 - Raw data used in phenotypic analysis and raw data for pollen viabilty used in analysis of means and variance. (PDF, 284 KB)
- Table S1 - Mean ± SE and samples sizes (in parentheses) for 2C DNA content as measured by flow cytometry from diploid (2x) and synthetic polyploid lines (4x) used in the experiment. (PDF, 400 KB)
- Table S2 - Principal components results using all floral traits, excluding the S2 and S4 data sets. (PDF, 287 KB)
- Table S3 - P-values for a Shapiro-Wilk W test for a goodness of fit for normal distribution on the first line, and if significantly different from normal distribution (p-value <= .05), tested for goodness-of-fit to lognormal distribution using Kolmogorov's D via the JMP Distribution function. (PDF, 424 KB)
- Table S4 - Mean ± standard error for floral traits of all genotypic classes measured in the phenotypic analysis. (PDF, 320 KB)
- Table S5 - Means (first row) of each trait, and letters indicating significance of difference for each trait for the subclasses (second row). (PDF, 411 KB)
