# Supplementary material for: Near-Absent Levels of Segregational Variation Suggest Limited Opportunities for the Introduction of Genetic Variation Via Homeologous Chromosome Pairing in Synthetic Neoallotetraploid Mimulus
Source: G3 (Bethesda). 2014 Jan 27;4(3):509–22. doi: 10.1534/g3.113.008441 (PMC3962489; doi:10.1534/g3.113.008441)
Supplement: Supporting Information [file supp_g3.113.008441_008441SI.pdf]

G3 · Genes | Genomes | Genetics

Near-Absent Levels Of Segregational Variation Suggest Limited Opportunities For The Introduction Of Genetic Variation Via Homeologous Chromosome Pairing In Synthetic Neoallotetraploid *Mimulus*

Jennifer L. Modliszewski*[1,2] and John H. Willis*

***Department of Biology, Duke University, Durham, NC, USA 27708

[1]Present address: Department of Biology, The University of North Carolina at Chapel Hill, Chapel Hill, NC, USA 27599
[2]Corresponding author: Department of Biology, The University of North Carolina at Chapel Hill, Chapel Hill, NC, USA 27599. E-mail: jenmod@live.unc.edu

DOI: 10.1534/g3.113.008441

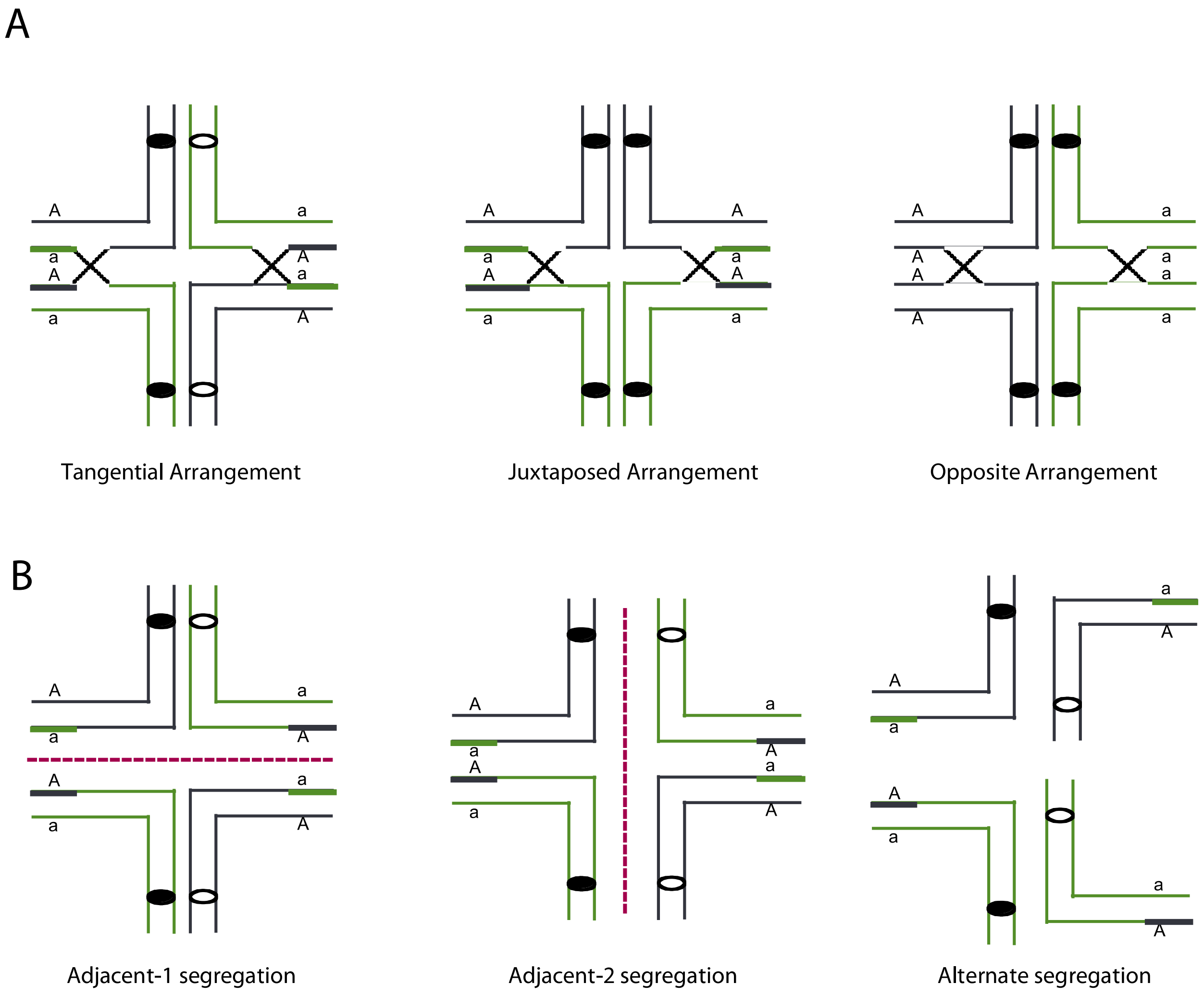

Figure S1 Quadrivalent arrangments (A) Three possible arrangements of chromosomes in a quadrivalent, with crossovers shown between the locus and the centromere (as an **X**). (B) Three possible segregation types in a quadrivalent, shown for the tangential arrangement. Chromosomes from Species 1 are shown in black and chromosomes from Species 2 are shown in green. Centromeres are marked with an oval, with centromeres of the tangential arrangement shown as filled or open to facilitate understanding of the modes of segregation. Species 1 has only 'A' alleles and Species 2 has only 'a' alleles. A magenta dashed line indicates the division of cells at Meiosis I for the adjacent segregations.

File S1

Raw data used in phenotypic analysis and raw data for pollen viabilty used in analysis of means and variance

Available for download at http://dx.doi.org/10.6084/m9.figshare.904927

Table S1 Mean ± SE and samples sizes (in parentheses) for 2C DNA content as measured by flow cytometry from diploid (2x) and synthetic polyploid lines (4x) used in the experiment.

Class	2C DNA content (pg)
IM-2x (n=1)	1.08[a]
SF-2x (n=1)	0.923[a]
F_1-2x (n=1)	0.99[a]
IM-4x (n=4)	2.18 ± 0.019
SF-4x (n=4)	1.90 ± 0.005
F_1-4x (n=7)	2.04 ± 0.018
F_2-4x (n=16)	2.00 ± 0.007
S_2 (n=21)[b]	2.00 ± 0.004
S_4-G (n=6)	2.01 ± 0.021
S_4-N (n=6)	2.04 ± 0.08

Footnotes.

a. If no SE is given, only one individual was measured.
b. The S_2 class consisted of 21 groups of pooled individuals.

Table S2 Principal components results using all floral traits, excluding the S_2 and S_4 data sets

**Principal Component**	**1**	**2**
Eigenvalue	6.9485	0.902
Percent variation explained	77.21	10.02
p-value	<.0001	<.0001
Loading		
Tube width	0.92435	0.01524
Tube length	0.89243	-0.28836
Corolla width	0.96381	0.09771
Corolla length	0.97848	-0.00855
Stamen length	0.83175	0.31816
Pistil length	0.85688	-0.468
Stigma-anther separation	0.96325	0.00942
Corolla width:tube length ratio	0.62902	0.67718
Lower calyx width	0.81348	-0.17358

Table S3 P-values for a Shapiro-Wilk W test for a goodness of fit for normal distribution on the first line, and if significantly different from normal distribution (p-value <= .05), tested for goodness-of-fit to lognormal distribution using Kolmogorov's D via the JMP Distribution function.

Trait		IM-4x	IM-2x	F_1-4x	F_1-2x	F_2-4x	F_{2G}-4x	F_{2N}-4x	F_2-2x	F_{2G}-2x	F_{2N}-2x	SF-2x	SF-4x	FAN	ROG
FT[a]	Normal[b]	0.070	**<0.0001**	**<0.0001**	**<0.0001**	**<0.0001**	**<0.0001**	**<0.0001**	**<0.0001**	**<0.0001**	**<0.0001**	**<0.0001**	**<0.0001**	**<0.0001**	0.489
	Log-normal[b]		**0.010**	**0.010**	**0.010**	**0.010**	**0.010**	**0.010**	**0.010**	**0.010**	**0.010**	**0.010**	**0.010**	**0.010**	
TW	Normal	0.062	**0.020**	0.832	0.168	**0.004**	**0.014**	0.061	**0.006**	**0.008**	0.2008	**0.041**	**<0.0001**	0.058	0.079
	Log-normal		**0.010**			**0.010**	**0.010**		**0.010**	**0.033**		**0.010**	**0.010**		
TL	Normal	0.122	**0.021**	0.119	0.463	**0.023**	0.097	**0.043**	**0.006**	0.513	**0.001**	**0.002**	**<0.0001**	0.253	0.243
	Log-normal		**0.010**			**0.010**		**0.010**	**0.010**		**0.010**	**0.010**	**0.010**		
CW	Normal	0.643	0.211	0.474	0.877	**<0.0001**	**0.000**	**0.022**	**0.020**	**0.007**[c]	**0.014**	**0.006**	**<0.0001**	0.264	0.485
	Log-normal					**0.010**	**0.010**	**0.010**	**0.010**	0.150	**0.010**	**0.010**	**0.010**		
CL	Normal	0.391	0.383	0.508	0.284	**<0.0001**	**0.002**	**0.003**	**<0.0001**	0.935	**<0.0001**	**0.046**	**<0.0001**	0.221	0.783
	Log-normal					**0.010**	**0.010**	**0.010**	**0.010**		**0.010**	**0.025**	**0.010**		
SL	Normal	**0.002**	0.055	0.166	0.060	**<0.0001**	**0.003**	**0.013**	**0.009**	**0.048**	**0.000**	**0.011**	**<0.0001**	**0.003**	0.168
	Log-normal	**0.010**				**0.010**	**0.010**	**0.010**	**0.010**	**0.010**	**0.010**	**0.010**	**0.010**	0.053	
PL	Normal	**<0.0001**	0.095	0.576	0.319	**<0.0001**	**0.000**	**0.024**	**0.001**	0.235	**0.000**	**<0.0001**	**<0.0001**	**0.002**	0.444
	Log-normal	**0.010**				**0.010**	**0.010**	**0.010**	**0.010**		**0.010**	**0.010**	**0.010**	**0.010**	
SAS	Normal	**<0.0001**	0.062	0.371	0.145	**<0.0001**	**<0.0001**	**0.018**	**<0.0001**	0.093	**0.004**	**<0.0001**	0.3483	**0.010**	0.198
	Log-normal	NA[d]				NA	NA	NA	NA		NA	NA		NA	
WLR	Normal	0.137	0.616	**0.043**	**0.000**	**<0.0001**	**0.001**	**0.016**	**0.006**	**0.036**	**0.016**	**0.001**	**<0.0001**	0.114	0.336
	Log-normal			**0.032**	0.130	**0.010**	**0.010**	0.150	**0.037**	0.150	**0.039**	**0.010**	**0.010**		
LXW	Normal	0.203	**<0.0001**	0.522	0.297	**0.003**	**0.040**	**0.001**	**<0.0001**	**<0.0001**	0.265	0.322	**<0.0001**	**<0.0001**	**0.002**
	Log-normal		**0.010**			**0.010**	**0.010**	**0.010**	**0.010**	**0.010**			**0.010**	**0.010**	**0.010**
PC1	Normal	**0.007**	0.512	0.905	0.446	**0.001**	**0.013**	**0.018**	**0.000**	0.150	**<0.0001**	**0.039**	**<0.0001**	**0.071**	0.981
	Log-normal	NA[d]				NA	NA	NA	NA		NA	NA	NA	NA	
PC2	Normal	**0.032**	0.617	0.852	0.315	**<0.0001**	**<0.0001**	**0.008**	**0.005**	0.347	0.336	**0.002**	0.636	**0.003**	0.880
	Log-normal	NA[d]				NA	NA	NA	NA			NA		NA	

Table S3, continued.

Footnotes:

a. Abbreviations used: FT = flowering time, TW = tube width, TL= tube length, CW = corolla width, CL = corolla length, WLR = tube width: corolla length ratio, SL = stamen length, PL = carpel (pistil) length, SAS = stigma-anther separation, LXW = lower calyx width, PC1 = principal component 1, PC2 = principal component 2.
b. Distributions differing significantly from the normal distribution (or log-normal distribution, when appropriate) are indicated in bold font.
c. Traits that fit a log-normal distribution significantly better than a normal distribution are indicated with a grey box.
d. A log-normal distribution cannot be test for those traits with negative values; these instances are indicated with 'NA'.

Table S4 Mean ± standard error for floral traits of all genotypic classes measured in the phenotypic analysis. Sample size is indicated in parentheses, and uppercase letters indicate statistically significant differences (p-value <= 0.05) within each trait among classes. Trait abbreviations are given in parentheses.

Trait	IM-4x	IM-2x	F_1-4x	F_1-2x	F_2-4x	F_2-2x	S_2-G	S_2-N	SF-4x	SF-2x	FAN	ROG
Flowering time (FT)	36.12 ± 0.627 (100) A	27.53 ± 0.328 (100) B	26.392 ± 0.510 (51) BC	23.151 ± 0.187 (86) D	26.219 ± 0.189 (342) BC	25.471 ± 0.240 (297) C	27.228 ± 0.371 (145) B	25.700 ± 0.297 (70) BC	27.260 ± 0.510 (100) B	22.343 ± 0.181 (67) D	27.897 ± 0.719 (39) B	22.357 ± 0.422 (28) D
Tube width (TW)	10.509 ± 0.105 (99) A	9.312 ± 0.083 (100) BC	9.836 ± 0.174 (51) AB	7.756 ± 0.098 (86) D	8.887 ± 0.075 (341) C	7.460 ± 0.078 (299) D	9.482 ± 0.099 (145) B	9.369 ± 0.143 (70) BC	2.473 ± 0.120 (98) G	3.587 ± 0.124 (66) F	5.243 ± 0.130 (39) E	3.946 ± 0.129 (28) F
Tube length (TL)	13.103 ± 0.112 (99) DE	12.908 ± 0.085 (100) E	14.926 ± 0.179 (51) A	14.008 ± 0.123 (86) BC	13.901 ± 0.084 (341) C	13.455 ± 0.081 (299) D	14.501 ± 0.106 (145) AB	14.652 ± 0.149 (70) AB	8.364 ± 0.164 (100) H	10.156 ± 0.141 (66) F	9.222 ± 0.153 (39) G	7.829 ± 0.180 (28) H
Corolla width (CW)	28.102 ± 0.251 (99) A	27.770 ± 0.227 (100) A	25.808 ± 0.386 (51) B	22.689 ± 0.264 (86) DE	23.731 ± 0.186 (341) CD	22.063 ± 0.231 (299) E	24.822 ± 0.245 (145) B	25.026 ± 0.279 (70) BC	4.559 ± 0.288 (100) H	7.836 ± 0.348 (66) G	11.840 ± 0.267 (39) F	8.781 ± 0.300 (28) G
Corolla length (CL)	29.297 ± 0.243 (99) A	28.265 ± 0.155 (100) AB	28.827 ± 0.345 (51) AB	26.323 ± 0.214 (85) CD	27.104 ± 0.162 (341) C	26.157 ± 0.185 (299) D	28.070 ± 0.209 (145) B	28.488 ± 0.255 (70) AB	10.996 ± 0.328 (100) G	14.948 ± 0.359 (66) EF	15.728 ± 0.247 (39) E	13.099 ± 0.289 (28) F
Stamen length (SL)	12.687 ± 0.104 (99) D	12.517 ± 0.057 (100) D	14.652 ± 0.134 (51) A	13.542 ± 0.089 (86) C	14.012 ± 0.064 (341) B	12.381 ± 0.078 (299) D	14.518 ± 0.085 (145) A	14.558 ± 0.098 (70) A	8.966 ± 0.158 (100) G	9.652 ± 0.134 (66) F	10.421 ± 0.148 (39) E	8.772 ± 0.177 (28) G
Pistil length (PL)	16.318 ± 0.152 (99) B	15.584 ± 0.073 (99) C	17.327 ± 0.170 (51) A	15.326 ± 0.092 (86) CD	16.348 ± 0.076 (341) B	14.993 ± 0.090 (299) D	16.753 ± 0.107 (145) AB	17.098 ± 0.113 (70) A	9.111 ± 0.180 (100) E	9.478 ± 0.136 (67) E	9.750 ± 0.146 (39) E	8.827 ± 0.186 (28) E

Stigma-anther separation	3.630 ± 0.107	3.066 ± 0.055	2.674 ± 0.090	1.784 ± 0.072	2.336 ± 0.043	2.612 ± 0.075	2.235 ± 0.056	2.540 ± 0.079	0.145 ± 0.069	-0.177 ± 0.120	-0.671 ± 0.095	0.054 ± 0.111
	(99)	(99)	(51)	(86)	(341)	(299)	(145)	(70)	(100)	(66)	(39)	(28)
(SAS)	A	B	BCD	E	D	C	D	CD	F	FG	G	FG
Corolla width:length ratio	2.151 ± 0.017	2.154 ± 0.016	1.728 ± 0.015	1.619 ± 0.012	1.704 ± 0.008	1.635 ± 0.013	1.709 ± 0.008	1.710 ± 0.012	0.518 ± 0.020	0.755 ± 0.028	1.284 ± 0.019	1.119 ± 0.023
	(99)	(100)	(51)	(85)	(341)	(299)	(145)	(70)	(100)	(66)	(39)	(28)
(WLR)	A	A	B	C	B	C	B	B	G	F	D	E
Lower calyx width	6.664 ± 0.103	4.763 ± 0.048	6.709 ± 0.118	4.762 ± 0.087	6.010 ± 0.060	5.105 ± 0.082	6.401 ± 0.084	6.534± 0.120	3.084 ± 0.135	3.987 ± 0.134	2.787 ± 0.076	1.195 ± 0.120
	(98)	(100)	(51)	(75)	(340)	(278)	(145)	(69)	(100)	(43)	(38)	(27)
(LXW)	A	C	A	C	B	C	A	A	E	D	E	F
Principal component 1	2.198 ± 0.101	1.429 ± 0.059	2.311 ± 0.143	0.630 ± 0.087	1.404 ± 0.068	0.395 ± 0.073	1.886 ± 0.094	2.043 ± 0.107	-5.596 ± 0.127	-4.385 ± 0.127	-3.742 ± 0.112	-5.123 ± 0.134
	(100)	(100)	(51)	(86)	(342)	(299)	(145)	(70)	(100)	(67)	(39)	(28)
(PC1)	A	BC	A	D	C	D	AB	A	G	EF	E	FG
Principal component 2	1.068 ± 0.074	0.889 ± 0.042	-0.127 ± 0.059	-0.403 ± 0.059	-0.144 ± 0.034	0.344 ± 0.055	-0.360 ± 0.040	-0.176 ± 0.065	-0.722 ± 0.055	-1.145 ± 0.095	-1.263 ± 0.082	-0.366 ± 0.090
	(100)	(100)	(51)	(86)	(342)	(299)	(145)	(70)	(100)	(67)	(39)	(28)
(PC2)	A	A	C	CD	C	B	C	C	D	E	E	CD
Percent viable pollen	0.680 ± 0.037	0.881 ± 0.016	0.827 ± 0.030	0.645 ± 0.025	0.930 ± 0.008	0.650 ± 0.030	0.956 ± 0.009	0.952 ± 0.009	0.931 ± 0.015	0.962 ± 0.016	0.987 ± 0.004	0.987 ± 0.003
	(20)	(21)	(41)	(44)	(103)	(91)	(52)	(50)	(36)	(19)	(20)	(26)
(VIAP)	C	AB	B	C	A	C	A	A	AB	AB	A	A

Table S5 Means (first row) of each trait, and letters indicating significance of difference for each trait for the subclasses (second row). For each pair (e.g., F_{1g}-2x and F_{1n}-2x) the letters shown only refer to whether or not the means of that pair are significantly different from one another; pairs with values that are significantly different from one another are indicated in bold font. *guttatus* and *nasutus* refer to the maternal parent used in the cross. Abbreviations used: FT = flowering time, TW = tube width, TL= tube length, CW = corolla width, CL = corolla length, WLR = tube width: corolla length ratio, SL = stamen length, PL = carpel (pistil) length, SAS = stigma-anther separation, LXW = lower calyx width, PC1 = principal component 1, PC2 = principal component 2.

	**F_1-4x**		**F_1-2x**		**F_2-4x**		**F_2-2x**	
**Trait**	*guttatus*	*nasutus*	*guttatus*	*nasutus*	*guttatus*	*nasutus*	*guttatus*	*nasutus*
FT	28.381	25.000	23.806	22.680	26.862	25.554	25.327	25.613
	A	A	A	A	A	A	A	A
TW	10.172	9.601	7.528	7.920	8.852	8.923	7.364	7.553
	A	A	A	A	A	A	A	A
TL	15.325	14.647	13.533	14.351	**13.560**	**14.251**	13.578	13.337
	A	A	A	A	**A**	**B**	A	A
CW	26.017	25.662	22.027	23.165	**22.870**	**24.618**	22.328	21.807
	A	A	A	A	**A**	**B**	A	A
CL	29.041	28.677	25.668	26.805	**26.444**	**27.783**	26.404	25.918
	A	A	A	A	**A**	**B**	A	A
WLR	1.695	1.752	1.627	1.614	1.681	1.727	1.639	1.632
	A	A	A	A	A	A	A	A
SL	14.865	14.503	13.286	13.726	13.795	14.235	**12.736**	**12.037**
	A	A	A	A	A	A	**A**	**B**
PL	17.647	17.103	15.162	15.443	16.190	16.510	14.965	15.019
	A	A	A	A	A	A	A	A
SAS	2.782	2.599	1.877	1.717	2.395	2.275	**2.229**	**2.983**
	A	A	A	A	A	A	**A**	**B**
LXW	6.689	6.723	4.490	4.987	**5.705**	**6.323**	5.036	5.173
	A	A	A	A	**A**	**B**	A	A
PC1	2.505	2.176	0.356	0.828	**1.146**	**1.670**	0.366	0.425
	A	A	A	A	**A**	**B**	A	A
PC2	-0.125	-0.128	-0.239	-0.521	-0.034	-0.257	**0.644**	**0.033**
	A	A	A	A	A	A	**A**	**B**
