# Supplementary material for: Near-Absent Levels of Segregational Variation Suggest Limited Opportunities for the Introduction of Genetic Variation Via Homeologous Chromosome Pairing in Synthetic Neoallotetraploid Mimulus
Source: G3 (Bethesda). 2014 Jan 27;4(3):509–22. doi: 10.1534/g3.113.008441 (PMC3962489; doi:10.1534/g3.113.008441)
Supplement: Supporting Information [file supp_g3.113.008441_FigureS1.pdf]

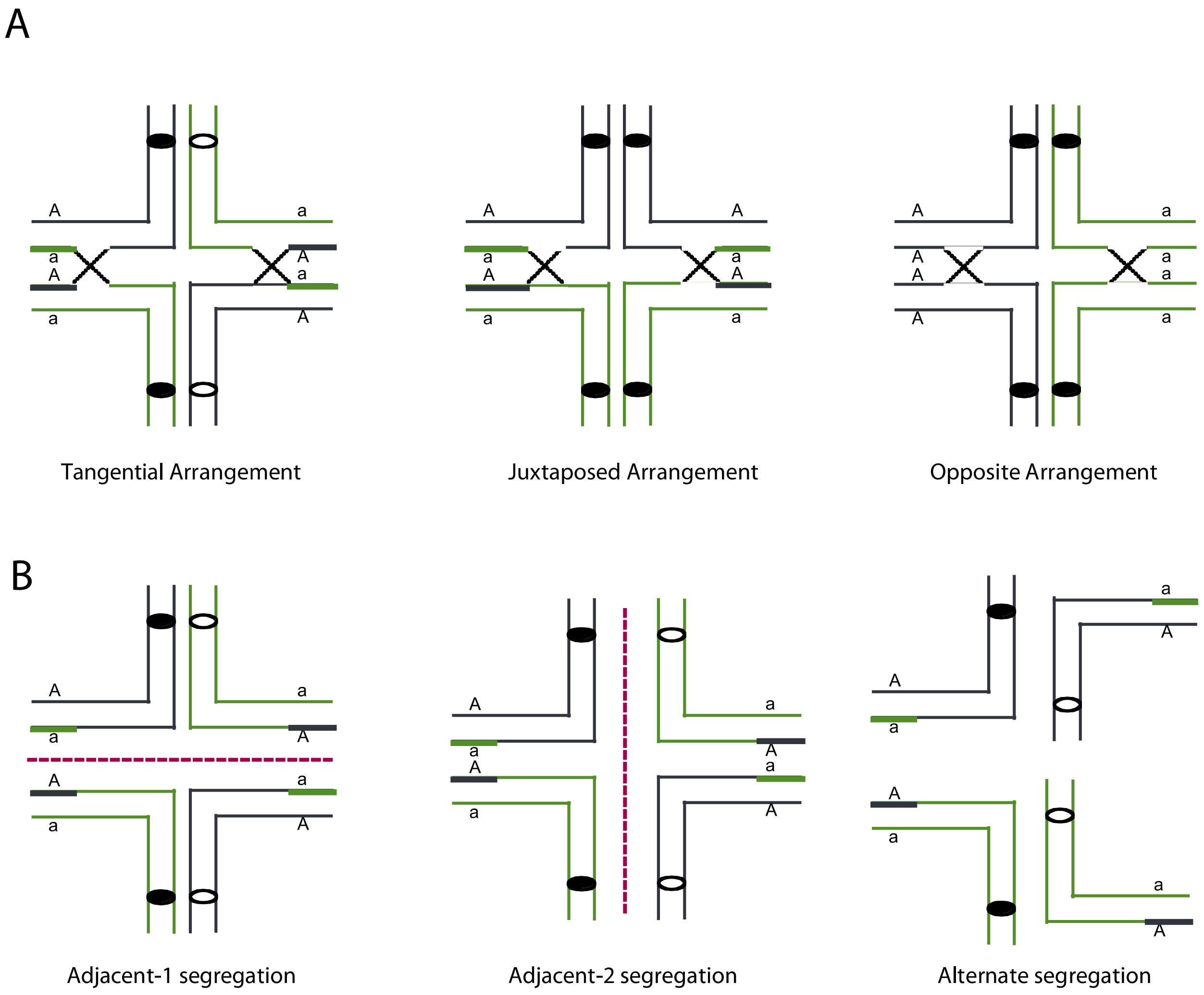

Figure S1 Quadrivalent arrangments (A) Three possible arrangements of chromosomes in a quadrivalent, with crossovers shown between the locus and the centromere (as an **X**). (B) Three possible segregation types in a quadrivalent, shown for the tangential arrangement. Chromosomes from Species 1 are shown in black and chromosomes from Species 2 are shown in green. Centromeres are marked with an oval, with centromeres of the tangential arrangement shown as filled or open to facilitate understanding of the modes of segregation. Species 1 has only 'A' alleles and Species 2 has only 'a' alleles. A magenta dashed line indicates the division of cells at Meiosis I for the adjacent segregations.
