# Supplementary material for: Near-Absent Levels of Segregational Variation Suggest Limited Opportunities for the Introduction of Genetic Variation Via Homeologous Chromosome Pairing in Synthetic Neoallotetraploid Mimulus
Source: G3 (Bethesda). 2014 Jan 27;4(3):509–22. doi: 10.1534/g3.113.008441 (PMC3962489; doi:10.1534/g3.113.008441)
Supplement: Supporting Information [file supp_g3.113.008441_FileS1.pdf]

File S1

Raw data used in phenotypic analysis and raw data for pollen viabilty used in analysis of means and variance

Available for download at http://dx.doi.org/10.6084/m9.figshare.904927
