# Supplementary material for: Near-Absent Levels of Segregational Variation Suggest Limited Opportunities for the Introduction of Genetic Variation Via Homeologous Chromosome Pairing in Synthetic Neoallotetraploid Mimulus
Source: G3 (Bethesda). 2014 Jan 27;4(3):509–22. doi: 10.1534/g3.113.008441 (PMC3962489; doi:10.1534/g3.113.008441)
Supplement: Supporting Information [file supp_g3.113.008441_TableS1.pdf]

Table S1 Mean ± SE and samples sizes (in parentheses) for 2C DNA content as measured by flow cytometry from diploid (2x) and synthetic polyploid lines (4x) used in the experiment.

Class	2C DNA content (pg)
IM-2x (n=1)	1.08[a]
SF-2x (n=1)	0.923[a]
F_1-2x (n=1)	0.99[a]
IM-4x (n=4)	2.18 ± 0.019
SF-4x (n=4)	1.90 ± 0.005
F_1-4x (n=7)	2.04 ± 0.018
F_2-4x (n=16)	2.00 ± 0.007
S_2 (n=21)[b]	2.00 ± 0.004
S_4-G (n=6)	2.01 ± 0.021
S_4-N (n=6)	2.04 ± 0.08

Footnotes.

a. If no SE is given, only one individual was measured.
b. The S_2 class consisted of 21 groups of pooled individuals.
