# Supplementary material for: Near-Absent Levels of Segregational Variation Suggest Limited Opportunities for the Introduction of Genetic Variation Via Homeologous Chromosome Pairing in Synthetic Neoallotetraploid Mimulus
Source: G3 (Bethesda). 2014 Jan 27;4(3):509–22. doi: 10.1534/g3.113.008441 (PMC3962489; doi:10.1534/g3.113.008441)
Supplement: Supporting Information [file supp_g3.113.008441_TableS2.pdf]

Table S2 Principal components results using all floral traits, excluding the S_2 and S_4 data sets

**Principal Component**	**1**	**2**
Eigenvalue	6.9485	0.902
Percent variation explained	77.21	10.02
p-value	<.0001	<.0001
Loading		
Tube width	0.92435	0.01524
Tube length	0.89243	-0.28836
Corolla width	0.96381	0.09771
Corolla length	0.97848	-0.00855
Stamen length	0.83175	0.31816
Pistil length	0.85688	-0.468
Stigma-anther separation	0.96325	0.00942
Corolla width:tube length ratio	0.62902	0.67718
Lower calyx width	0.81348	-0.17358
