# Supplementary material for: Near-Absent Levels of Segregational Variation Suggest Limited Opportunities for the Introduction of Genetic Variation Via Homeologous Chromosome Pairing in Synthetic Neoallotetraploid Mimulus
Source: G3 (Bethesda). 2014 Jan 27;4(3):509–22. doi: 10.1534/g3.113.008441 (PMC3962489; doi:10.1534/g3.113.008441)
Supplement: Supporting Information [file supp_g3.113.008441_TableS3.pdf]

Table S3 P-values for a Shapiro-Wilk W test for a goodness of fit for normal distribution on the first line, and if significantly different from normal distribution (p-value <= .05), tested for goodness-of-fit to lognormal distribution using Kolmogorov's D via the JMP Distribution function.

Trait		IM-4x	IM-2x	F_1-4x	F_1-2x	F_2-4x	F_{2G}-4x	F_{2N}-4x	F_2-2x	F_{2G}-2x	F_{2N}-2x	SF-2x	SF-4x	FAN	ROG
FT[a]	Normal[b]	0.070	**<0.0001**	**<0.0001**	**<0.0001**	**<0.0001**	**<0.0001**	**<0.0001**	**<0.0001**	**<0.0001**	**<0.0001**	**<0.0001**	**<0.0001**	**<0.0001**	0.489
	Log-normal[b]		**0.010**	**0.010**	**0.010**	**0.010**	**0.010**	**0.010**	**0.010**	**0.010**	**0.010**	**0.010**	**0.010**	**0.010**	
TW	Normal	0.062	**0.020**	0.832	0.168	**0.004**	**0.014**	0.061	**0.006**	**0.008**	0.2008	**0.041**	**<0.0001**	0.058	0.079
	Log-normal		**0.010**			**0.010**	**0.010**		**0.010**	**0.033**		**0.010**	**0.010**		
TL	Normal	0.122	**0.021**	0.119	0.463	**0.023**	0.097	**0.043**	**0.006**	0.513	**0.001**	**0.002**	**<0.0001**	0.253	0.243
	Log-normal		**0.010**			**0.010**		**0.010**	**0.010**		**0.010**	**0.010**	**0.010**		
CW	Normal	0.643	0.211	0.474	0.877	**<0.0001**	**0.000**	**0.022**	**0.020**	**0.007**[c]	**0.014**	**0.006**	**<0.0001**	0.264	0.485
	Log-normal					**0.010**	**0.010**	**0.010**	**0.010**	0.150	**0.010**	**0.010**	**0.010**		
CL	Normal	0.391	0.383	0.508	0.284	**<0.0001**	**0.002**	**0.003**	**<0.0001**	0.935	**<0.0001**	**0.046**	**<0.0001**	0.221	0.783
	Log-normal					**0.010**	**0.010**	**0.010**	**0.010**		**0.010**	**0.025**	**0.010**		
SL	Normal	**0.002**	0.055	0.166	0.060	**<0.0001**	**0.003**	**0.013**	**0.009**	**0.048**	**0.000**	**0.011**	**<0.0001**	**0.003**	0.168
	Log-normal	**0.010**				**0.010**	**0.010**	**0.010**	**0.010**	**0.010**	**0.010**	**0.010**	**0.010**	0.053	
PL	Normal	**<0.0001**	0.095	0.576	0.319	**<0.0001**	**0.000**	**0.024**	**0.001**	0.235	**0.000**	**<0.0001**	**<0.0001**	**0.002**	0.444
	Log-normal	**0.010**				**0.010**	**0.010**	**0.010**	**0.010**		**0.010**	**0.010**	**0.010**	**0.010**	
SAS	Normal	**<0.0001**	0.062	0.371	0.145	**<0.0001**	**<0.0001**	**0.018**	**<0.0001**	0.093	**0.004**	**<0.0001**	0.3483	**0.010**	0.198
	Log-normal	NA[d]				NA	NA	NA	NA		NA	NA		NA	
WLR	Normal	0.137	0.616	**0.043**	**0.000**	**<0.0001**	**0.001**	**0.016**	**0.006**	**0.036**	**0.016**	**0.001**	**<0.0001**	0.114	0.336
	Log-normal			**0.032**	0.130	**0.010**	**0.010**	0.150	**0.037**	0.150	**0.039**	**0.010**	**0.010**		
LXW	Normal	0.203	**<0.0001**	0.522	0.297	**0.003**	**0.040**	**0.001**	**<0.0001**	**<0.0001**	0.265	0.322	**<0.0001**	**<0.0001**	**0.002**
	Log-normal		**0.010**			**0.010**	**0.010**	**0.010**	**0.010**	**0.010**			**0.010**	**0.010**	**0.010**
PC1	Normal	**0.007**	0.512	0.905	0.446	**0.001**	**0.013**	**0.018**	**0.000**	0.150	**<0.0001**	**0.039**	**<0.0001**	**0.071**	0.981
	Log-normal	NA[d]				NA	NA	NA	NA		NA	NA	NA	NA	
PC2	Normal	**0.032**	0.617	0.852	0.315	**<0.0001**	**<0.0001**	**0.008**	**0.005**	0.347	0.336	**0.002**	0.636	**0.003**	0.880
	Log-normal	NA[d]				NA	NA	NA	NA			NA		NA	

Table S3, continued.

Footnotes:

a. Abbreviations used: FT = flowering time, TW = tube width, TL= tube length, CW = corolla width, CL = corolla length, WLR = tube width: corolla length ratio, SL = stamen length, PL = carpel (pistil) length, SAS = stigma-anther separation, LXW = lower calyx width, PC1 = principal component 1, PC2 = principal component 2.
b. Distributions differing significantly from the normal distribution (or log-normal distribution, when appropriate) are indicated in bold font.
c. Traits that fit a log-normal distribution significantly better than a normal distribution are indicated with a grey box.
d. A log-normal distribution cannot be test for those traits with negative values; these instances are indicated with 'NA'.
