# Supplementary material for: Near-Absent Levels of Segregational Variation Suggest Limited Opportunities for the Introduction of Genetic Variation Via Homeologous Chromosome Pairing in Synthetic Neoallotetraploid Mimulus
Source: G3 (Bethesda). 2014 Jan 27;4(3):509–22. doi: 10.1534/g3.113.008441 (PMC3962489; doi:10.1534/g3.113.008441)
Supplement: Supporting Information [file supp_g3.113.008441_TableS4.pdf]

Table S4 Mean ± standard error for floral traits of all genotypic classes measured in the phenotypic analysis. Sample size is indicated in parentheses, and uppercase letters indicate statistically significant differences (p-value <= 0.05) within each trait among classes. Trait abbreviations are given in parentheses.

Trait	IM-4x	IM-2x	F_1-4x	F_1-2x	F_2-4x	F_2-2x	S_2-G	S_2-N	SF-4x	SF-2x	FAN	ROG
Flowering time (FT)	36.12 ± 0.627 (100) A	27.53 ± 0.328 (100) B	26.392 ± 0.510 (51) BC	23.151 ± 0.187 (86) D	26.219 ± 0.189 (342) BC	25.471 ± 0.240 (297) C	27.228 ± 0.371 (145) B	25.700 ± 0.297 (70) BC	27.260 ± 0.510 (100) B	22.343 ± 0.181 (67) D	27.897 ± 0.719 (39) B	22.357 ± 0.422 (28) D
Tube width (TW)	10.509 ± 0.105 (99) A	9.312 ± 0.083 (100) BC	9.836 ± 0.174 (51) AB	7.756 ± 0.098 (86) D	8.887 ± 0.075 (341) C	7.460 ± 0.078 (299) D	9.482 ± 0.099 (145) B	9.369 ± 0.143 (70) BC	2.473 ± 0.120 (98) G	3.587 ± 0.124 (66) F	5.243 ± 0.130 (39) E	3.946 ± 0.129 (28) F
Tube length (TL)	13.103 ± 0.112 (99) DE	12.908 ± 0.085 (100) E	14.926 ± 0.179 (51) A	14.008 ± 0.123 (86) BC	13.901 ± 0.084 (341) C	13.455 ± 0.081 (299) D	14.501 ± 0.106 (145) AB	14.652 ± 0.149 (70) AB	8.364 ± 0.164 (100) H	10.156 ± 0.141 (66) F	9.222 ± 0.153 (39) G	7.829 ± 0.180 (28) H
Corolla width (CW)	28.102 ± 0.251 (99) A	27.770 ± 0.227 (100) A	25.808 ± 0.386 (51) B	22.689 ± 0.264 (86) DE	23.731 ± 0.186 (341) CD	22.063 ± 0.231 (299) E	24.822 ± 0.245 (145) B	25.026 ± 0.279 (70) BC	4.559 ± 0.288 (100) H	7.836 ± 0.348 (66) G	11.840 ± 0.267 (39) F	8.781 ± 0.300 (28) G
Corolla length (CL)	29.297 ± 0.243 (99) A	28.265 ± 0.155 (100) AB	28.827 ± 0.345 (51) AB	26.323 ± 0.214 (85) CD	27.104 ± 0.162 (341) C	26.157 ± 0.185 (299) D	28.070 ± 0.209 (145) B	28.488 ± 0.255 (70) AB	10.996 ± 0.328 (100) G	14.948 ± 0.359 (66) EF	15.728 ± 0.247 (39) E	13.099 ± 0.289 (28) F
Stamen length (SL)	12.687 ± 0.104 (99) D	12.517 ± 0.057 (100) D	14.652 ± 0.134 (51) A	13.542 ± 0.089 (86) C	14.012 ± 0.064 (341) B	12.381 ± 0.078 (299) D	14.518 ± 0.085 (145) A	14.558 ± 0.098 (70) A	8.966 ± 0.158 (100) G	9.652 ± 0.134 (66) F	10.421 ± 0.148 (39) E	8.772 ± 0.177 (28) G
Pistil length (PL)	16.318 ± 0.152 (99) B	15.584 ± 0.073 (99) C	17.327 ± 0.170 (51) A	15.326 ± 0.092 (86) CD	16.348 ± 0.076 (341) B	14.993 ± 0.090 (299) D	16.753 ± 0.107 (145) AB	17.098 ± 0.113 (70) A	9.111 ± 0.180 (100) E	9.478 ± 0.136 (67) E	9.750 ± 0.146 (39) E	8.827 ± 0.186 (28) E

Stigma-anther separation (SAS)	3.630 ± 0.107 (99) A	3.066 ± 0.055 (99) B	2.674 ± 0.090 (51) BCD	1.784 ± 0.072 (86) E	2.336 ± 0.043 (341) D	2.612 ± 0.075 (299) C	2.235 ± 0.056 (145) D	2.540 ± 0.079 (70) CD	0.145 ± 0.069 (100) F	-0.177 ± 0.120 (66) FG	-0.671 ± 0.095 (39) G	0.054 ± 0.111 (28) FG
Corolla width:length ratio (WLR)	2.151 ± 0.017 (99) A	2.154 ± 0.016 (100) A	1.728 ± 0.015 (51) B	1.619 ± 0.012 (85) C	1.704 ± 0.008 (341) B	1.635 ± 0.013 (299) C	1.709 ± 0.008 (145) B	1.710 ± 0.012 (70) B	0.518 ± 0.020 (100) G	0.755 ± 0.028 (66) F	1.284 ± 0.019 (39) D	1.119 ± 0.023 (28) E
Lower calyx width (LXW)	6.664 ± 0.103 (98) A	4.763 ± 0.048 (100) C	6.709 ± 0.118 (51) A	4.762 ± 0.087 (75) C	6.010 ± 0.060 (340) B	5.105 ± 0.082 (278) C	6.401 ± 0.084 (145) A	6.534± 0.120 (69) A	3.084 ± 0.135 (100) E	3.987 ± 0.134 (43) D	2.787 ± 0.076 (38) E	1.195 ± 0.120 (27) F
Principal component 1 (PC1)	2.198 ± 0.101 (100) A	1.429 ± 0.059 (100) BC	2.311 ± 0.143 (51) A	0.630 ± 0.087 (86) D	1.404 ± 0.068 (342) C	0.395 ± 0.073 (299) D	1.886 ± 0.094 (145) AB	2.043 ± 0.107 (70) A	-5.596 ± 0.127 (100) G	-4.385 ± 0.127 (67) EF	-3.742 ± 0.112 (39) E	-5.123 ± 0.134 (28) FG
Principal component 2 (PC2)	1.068 ± 0.074 (100) A	0.889 ± 0.042 (100) A	-0.127 ± 0.059 (51) C	-0.403 ± 0.059 (86) CD	-0.144 ± 0.034 (342) C	0.344 ± 0.055 (299) B	-0.360 ± 0.040 (145) C	-0.176 ± 0.065 (70) C	-0.722 ± 0.055 (100) D	-1.145 ± 0.095 (67) E	-1.263 ± 0.082 (39) E	-0.366 ± 0.090 (28) CD
Percent viable pollen (VIAP)	0.680 ± 0.037 (20) C	0.881 ± 0.016 (21) AB	0.827 ± 0.030 (41) B	0.645 ± 0.025 (44) C	0.930 ± 0.008 (103) A	0.650 ± 0.030 (91) C	0.956 ± 0.009 (52) A	0.952 ± 0.009 (50) A	0.931 ± 0.015 (36) AB	0.962 ± 0.016 (19) AB	0.987 ± 0.004 (20) A	0.987 ± 0.003 (26) A
