# Supplementary material for: Near-Absent Levels of Segregational Variation Suggest Limited Opportunities for the Introduction of Genetic Variation Via Homeologous Chromosome Pairing in Synthetic Neoallotetraploid Mimulus
Source: G3 (Bethesda). 2014 Jan 27;4(3):509–22. doi: 10.1534/g3.113.008441 (PMC3962489; doi:10.1534/g3.113.008441)
Supplement: Supporting Information [file supp_g3.113.008441_TableS5.pdf]

Table S5 Means (first row) of each trait, and letters indicating significance of difference for each trait for the subclasses (second row). For each pair (e.g., F_{1g}-2x and F_{1n}-2x) the letters shown only refer to whether or not the means of that pair are significantly different from one another; pairs with values that are significantly different from one another are indicated in bold font. *guttatus* and *nasutus* refer to the maternal parent used in the cross. Abbreviations used: FT = flowering time, TW = tube width, TL= tube length, CW = corolla width, CL = corolla length, WLR = tube width: corolla length ratio, SL = stamen length, PL = carpel (pistil) length, SAS = stigma-anther separation, LXW = lower calyx width, PC1 = principal component 1, PC2 = principal component 2.

	**F_1-4x**		**F_1-2x**		**F_2-4x**		**F_2-2x**	
**Trait**	*guttatus*	*nasutus*	*guttatus*	*nasutus*	*guttatus*	*nasutus*	*guttatus*	*nasutus*
FT	28.381	25.000	23.806	22.680	26.862	25.554	25.327	25.613
	A	A	A	A	A	A	A	A
TW	10.172	9.601	7.528	7.920	8.852	8.923	7.364	7.553
	A	A	A	A	A	A	A	A
TL	15.325	14.647	13.533	14.351	**13.560**	**14.251**	13.578	13.337
	A	A	A	A	**A**	**B**	A	A
CW	26.017	25.662	22.027	23.165	**22.870**	**24.618**	22.328	21.807
	A	A	A	A	**A**	**B**	A	A
CL	29.041	28.677	25.668	26.805	**26.444**	**27.783**	26.404	25.918
	A	A	A	A	**A**	**B**	A	A
WLR	1.695	1.752	1.627	1.614	1.681	1.727	1.639	1.632
	A	A	A	A	A	A	A	A
SL	14.865	14.503	13.286	13.726	13.795	14.235	**12.736**	**12.037**
	A	A	A	A	A	A	**A**	**B**
PL	17.647	17.103	15.162	15.443	16.190	16.510	14.965	15.019
	A	A	A	A	A	A	A	A
SAS	2.782	2.599	1.877	1.717	2.395	2.275	**2.229**	**2.983**
	A	A	A	A	A	A	**A**	**B**
LXW	6.689	6.723	4.490	4.987	**5.705**	**6.323**	5.036	5.173
	A	A	A	A	**A**	**B**	A	A
PC1	2.505	2.176	0.356	0.828	**1.146**	**1.670**	0.366	0.425
	A	A	A	A	**A**	**B**	A	A
PC2	-0.125	-0.128	-0.239	-0.521	-0.034	-0.257	**0.644**	**0.033**
	A	A	A	A	A	A	**A**	**B**
